# PRINCESS: comprehensive detection of haplotype resolved SNVs, SVs, and methylation

**DOI:** 10.1186/s13059-021-02486-w

**Published:** 2021-09-14

**Authors:** Medhat Mahmoud, Harshavardhan Doddapaneni, Winston Timp, Fritz J. Sedlazeck

**Affiliations:** 1 Human Genome Sequencing Center, Baylor College of Medicine, Houston, TX 77030 USA; 2 Department of Molecular and Human Genetics, Baylor College of Medicine, Houston, TX 77030 USA; 3 Department of Biomedical Engineering, Johns Hopkins University, Baltimore, MD 21218 USA

**Keywords:** Structural variant, Single-nucleotide variant, Methylation, Phasing, Oxford Nanopore, PacBio

## Abstract

Long-read sequencing has been shown to have advantages in structural variation (SV) detection and methylation calling. Many studies focus either on SV, methylation, or phasing of SNV; however, only the combination of variants provides a comprehensive insight into the sample and thus enables novel findings in biology or medicine. PRINCESS is a structured workflow that takes raw sequence reads and generates a fully phased SNV, SV, and methylation call set within a few hours. PRINCESS achieves high accuracy and long phasing even on low coverage datasets and can resolve repetitive, complex medical relevant genes that often escape detection. PRINCESS is publicly available at https://github.com/MeHelmy/princess under the MIT license.

**Supplementary Information:**

The online version contains supplementary material available at 10.1186/s13059-021-02486-w.

## Background

Long-read sequencing (LRS) is becoming more broadly available across sequencing centers and smaller academic institutions [1]. This is mainly driven by the availability of a variety of scalable instrumentation from Oxford Nanopore and Pacific Biosciences, but also by the improvements in yield, error rates (0.1–3%), and sample requirements [2, 3]. Current instruments allow the assessment of human genomes at unprecedented accuracy [4, 5] and scale [1, 6, 7]. LRS enables further investigation into many biological questions such as assessment of highly repetitive regions (e.g., *SMN1,2*) [8], resolving complex regions (e.g., *MHC, KIRR*) [9, 10], and improving our understanding of structural variants (SVs) [1, 7, 11]. More recent papers show that LRS enable a more detailed characterization of SVs especially over insertions [12, 13]. Previously, LRS was seen as too costly or erroneous, which several studies now show is no longer the case [1, 6, 7, 14]. Thus, LRS established its utility as one of the main components for genomic sequencing [15, 16]. Given these advancements, we see novel insights in human diseases [7, 17, 18], evolution [6, 14, 19], and other areas of biology and medical research [11].

The detection of small variants (SNVs and indels) (typically 1–50 bp), SVs (50+ bp: deletions, duplications, insertions, inversions, and translocations), and methylation differences provide important insights into genomics and genetics [20–22]. Each of these genomic variations/alterations have been shown to be important drivers of evolution, diversity, and diseases or phenotypic changes [6, 23, 24]. To detect these variations/alterations, multiple software methods have been introduced that focus either on de novo assembly [25, 26], mapping [27, 28], SNV calling [29, 30], SV calling [28, 31], SNV phasing [32], methylation calling [33, 34], quality assessment [35], and others [36]. Most often these methods require expert knowledge to tune the default parameters for different species or sequencing technologies. Furthermore, the results of these methods for LRS analysis often need to be filtered and sometimes even converted to be utilized by another program. Given the complexity of data analysis, recent studies focus on, e.g., SV identification [7] or methylation [34] and often ignore the other variants or haplotype signals. In addition, some applications are just limited. For example, all phasing methods currently operate on SNV and do not integrate larger insertion, deletions, or in general SVs [32]. There are now a few methods that can phase methylation, but again outside the context of SNV or SV phasing. Thus, we are producing long-read data sets, but are lacking methods to fully utilize them despite their higher cost for data generation and higher requirements on sample quality and quantity.

Here we present the first method to achieve accurate and haplotype resolved SNVs, indels, SVs, and methylation calls at scale with minimum coverage requirements: PRINCESS. PRINCESS consists of different modules that are managed by Snakemake [37] enabling straightforward adaptability to local machines, cluster, and cloud environments. Furthermore, PRINCESS implements several novel approaches to phase SVs and methylation signals given a single flow (Oxford Nanopore) or SMRT cell (Pacific Bioscience). In addition, PRINCESS automatically adapts itself to the underlying data, enabling its applications across different model and non-model species and technologies. PRINCESS achieves a high accuracy on SNV, SV, phasing of SNV together with SV, and integration of methylation results across low coverage PacBio High Fidelity (HiFi) or Oxford Nanopore reads. Thus, providing a comprehensive, haplotype resolved insight for each sample at hand at a minimum cost. Optionally, PRINCESS can also leverage the parental SNV to improve phasing further. We further demonstrate the versatility of PRINCESS across the whole genome and capture data. Lastly, we highlight PRINCESS’s capability to improve variant identification across 193 medical regions that are difficult to assess with short-read technology [38] that often escapes NGS sequencing [38].

## Results

### PRINCESS: an open framework for long-read variation detection and phasing

**Fig. 1:**
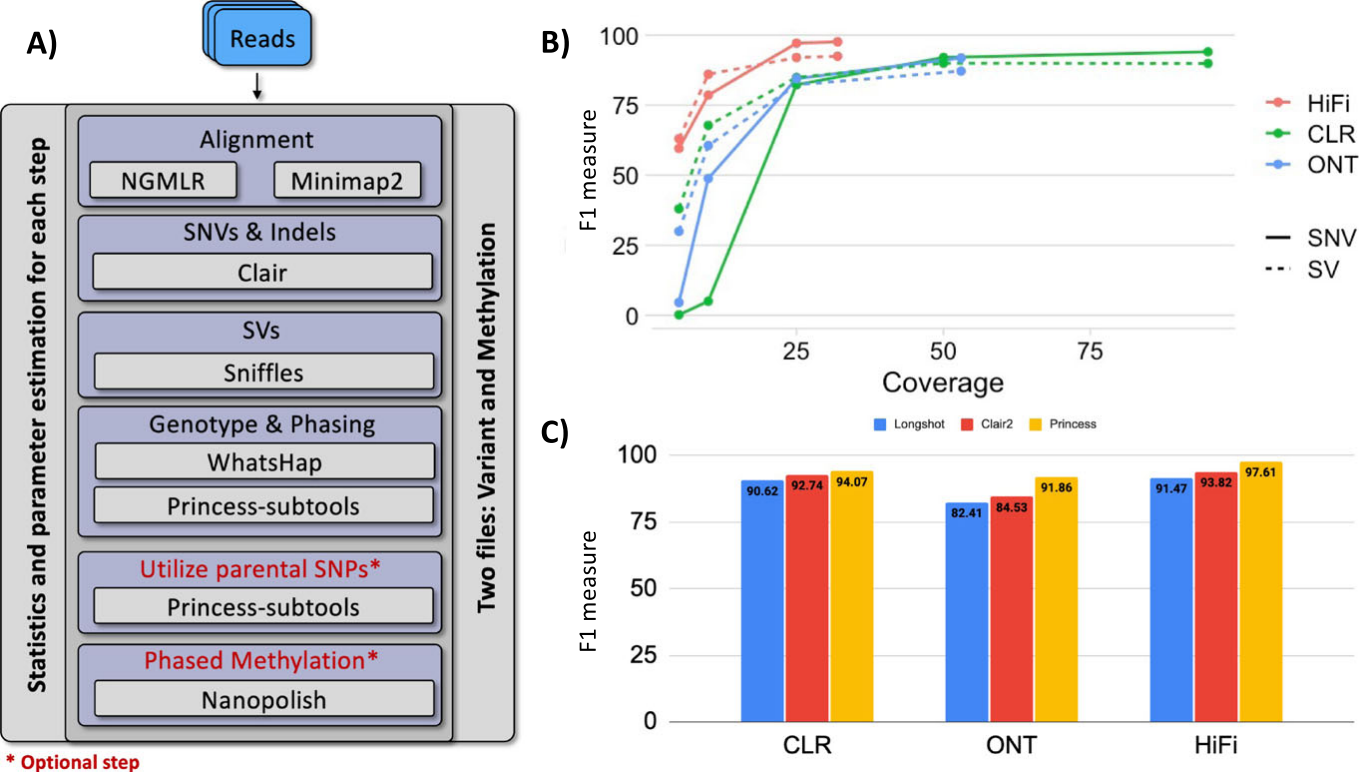
Overview of PRINCESS. **A** General workflow of PRINCESS from read input through phased variant calls, with optional steps indicated in red font. **B** F1-score Benchmarking results for SNVs, indels, and SVs based on HG002 GIAB gold standard across PacBio HiFi, PacBio Continuous Long Read (CLR), and ONT data. **C** Improved SNV calling of PRINCESS based on its automated SNV filtering compared to Clair2 [39] and Longshot [30]

PRINCESS uses raw reads and provides phased variants (SNVs, indels, and SVs) together with optional phased methylation. To achieve this, PRINCESS consists of multiple stages (see Fig. 1A) including (i) initial data quality control, (ii) alignment of the reads, (iii) identification of SNVs and indels, (iv) identification of SVs, (v) filtering variants, and (vi) phasing of SNVs, indels, and SVs together and (vii) reporting of the results (see “Methods”). To ease the use of PRINCESS, we have incorporated preset parameters to optimize the analysis of the three major long-read platforms/technologies being CLR, HiFi for PacBio, and Oxford Nanopore (ONT). For expert users, PRINCESS is highly configurable using a YAML file and also allows researchers to start or restart at intermediate steps (e.g., after mapping or using an existing SNVs and indels call set). Furthermore, each step includes summary reports to enable quality assessment of the results.

To highlight the performance of PRINCESS, we utilized the reference material from GIAB HG002 for SNVs, indels, and SVs based on different coverage levels and sequencing technologies [40, 41]. PRINCESS (Fig. 1C) implements an SNV filtering mechanism, which enhances the F1 score for all data sets (F1 measure from 93.81 to 97.60%) (Additional file 2: Table S1) and also improves in comparison to existing tools (e.g., Longshot [30]). For CLR and ONT, we increased F1 from 92.73 to 94.06% and from 84.53 to 91.85% respectively. Overall, PRINCESS achieves a high level of recall and precision (F-measure) across ONT (SNV: 91.85% SV:87.16%), PacBio CLR (SNV:94.06% SV:89.90%) and HiFi (SNV:97.60% SV:92.48%) for all variant types even when we down-sample the coverages to mimic one flow cell (HiFi: 10×, ONT, CLR: 25×) the performance remains high for ONT (SNV:84.45% SV:82.28%), PacBio CLR (SNV:82.29% SV:84.90%), and HiFi (SNV:78.58% SV:86.00%). Using PRINCESS, we reach a high genotyping accuracy (HiFi 99.81%, ONT 99.76%, and CLR 99.70%). Additional files 3, 4, 5: Tables S2-S4 have a detailed comparison across the different variation types and genotype performance of PRINCESS. Supplementary Section 1 describes the results in detail.

**Fig. 2:**
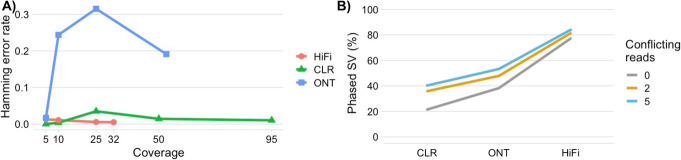
PRINCESS improvements for phasing: **A** Benchmarking results for phasing performance (Hamming error rate) for PRINCESS based on GIAB gold standard (the lower, the better). **B** Percentage of phased SVs across different conflicting read thresholds

Next, we evaluated SNVs and indel phasing, using WhatsHap [32] with the truth dataset, where we measured switch error (i.e., multiple SNVs or indels assigned to the incorrect haplotype) and Hamming error (i.e., total number of incorrectly assigned SNVs or indels to haplotypes). Figure 2A shows the overall Hamming error. The longest N50 is achieved by ONT (17,427 kbp) followed by CLR (151 kbp), and HiFi (117 kbp) respectively. Although ONT achieved the highest N50, it suffered from a high Hamming error rate (0.19) (see Fig. 2A), but with a switch error rate of 0.0036 similar to CLR data, which achieved a lower Hamming error of 0.01032. This highlights smaller inconsistencies (Hamming error) compared to large phase block errors (switch error rate). Lastly, HiFi data achieved the lowest switch (0.0040) and Hamming (0.0052) error rate (Additional file 6: Table S5 and Additional file 1: Figure S5). Overall the three technologies have very low switch error rates, but ONT secured the longest phasing N50 (Additional file 1: Figure S6) associated with a higher Hamming error rate than both HiFi and CLR data. Furthermore, we compared the performance of the three technologies using different coverage levels. Increasing the coverage for all technologies led to a lower switch and Hamming error, alongside an increase in N50 (Additional file 6: Table S5). Additionally, the HiFi insert size did not affect the SV precision rate. Increasing the insert size (25 k) with low coverage (11×) led to a minor reduction in F1 score for SNVs and indels, but increased the N50 from 140 to 250 kb (see Additional file 2: Table S1, Additional file 1: Figure S18). Interestingly, for phasing SNVs and indels without PRINCESS filtering, we observe a higher phasing error rates across all sequencing technologies, ranging from 0.0138 (HiFi) to 0.3609 (ONT) compared to 0.0103 (CLR) to 0.1912 (ONT) after filtering.

The deliverables from PRINCESS not only includes phased SNVs and indels, but further includes the first alignment approach for SV phasing and inclusion of methylation phasing. We observed the highest phase rate from HiFi (77.17%) followed by ONT (38.24%) and CLR (21.44%) (see Fig. 2B). The lower phasing rate of ONT and CLR is due to multiple reads that are in conflict with the phasing information for the SV. This might be also because the SNV calling and phasing in proximity to SV is often disturbed [42]. The majority of SVs that were phased are deletions followed by insertions HiFi (DEL: 84.72% and INS: 74.60%) and CLR (DEL: 33.54% and INS: 12.49%). For ONT, the insertions (48.60%) are the most phased SV type, followed by deletions (32.11%). Inversions (HiFi: 30.00%, ONT: 28.57%, and CLR 25.71%) and duplications (HiFi: 34.62%, ONT 32%, and CLR: 11.76%) showed a lower phasing rate, likely due to their size and complexity. PRINCESS was also able to phase translocations across CLR (51.11%), ONT (48.78%) and HiFi (23.02%) data.

Next, we investigated how SV phasing performance changed if we allow for 2 or 5 conflicting reads (user definable parameter). Figure 2B shows the overall phasing improvement of the SV with respect to the conflicting thresholds**.** The largest improvements are for CLR, as the data changed from 21.44% phased SV to 40.28%. This is followed by ONT from 38.24% up to 53.25% and HiFi 77.17% up to 83.97%. The largest gains were observed for deletions and insertions respectively across HiFi (DEL: 90.37%, INS: 81.88%), ONT (DEL: 50.12%, INS: 58.43%), and CLR (DEL: 44.33%, INS: 37.21%). For rearrangements, we observed a higher phasing rate for CLR (45.71% inversions, and 71.11% breakpoint notation (BNDs)) and ONT (60.00% inversions, and 68.29% BNDs) compared to HiFi data (40% inversions, and 38.13% BNDs).

Overall, PRINCESS shows a high rate of accuracy for the identification of SNVs and SVs and is further able to phase both variant types together. Thus, improving the insights gained into the sample at hand independent of the sequencing technology.

### Applying PRINCESS to capture data

So far we have demonstrated that PRINCESS is highly accurate in detecting and phasing SNVs, indels, and SVs. We have also assessed the performance of PRINCESS on recently published Cas9-based targeted data [43] using ONT MinION and Flongle sequencers. Our dataset includes 10 regions, across two non-tumorigenic cell lines (GM12878 and MCF-10A) and two cancer cell lines (MCF-7, MDA-MB-231).

We first assess the performance of PRINCESS for SNVs and indels identification with the previously published results. For GM12878 using ONT MinION data, PRINCESS shows a high sensitivity (87.44%) when comparing the 226 SNVs and indels identified with the GIAB NA12878 truth set. The sensitivity increases to 94.80% when only considering SNVs. Across the ten regions sequenced, we could phase 96.12% (99/103) of all the SNVs and indels. When running PRINCESS on the Flongle data, we observed a slightly lower sensitivity (80.40% for all variants (SNVs and indels), 83.82% for SNVs only) likely based on the drop of coverage identifying only 183 SNVs. Here, PRINCESS was able to phase 97.87% of the heterozygous SNVs and indels. Similarly, for MCF-10A, we identified 196 variants (169 SNVs and 27 indels). PRINCESS was able to phase 83.70% (113/135) heterozygous SNVs and indels.

For cancer samples, we compared the performance of PRINCESS to the previous variant calls. For MDA-MB-231, PRINCESS identified all 37 SNVs and indels that were identified from the previous study and was able to phase all heterozygous SNVs and indels (16). Similarly, for MCF-7, PRINCESS identified 147 SNVs and indels 98.46% of heterozygous SNVs and indels are phased (64/65), 128 SNVs and indels (87.07%) agree with the previously established call set, 97.87% phased (46/47).

Next, we investigated the concordance of our SV call set with the reported results for MDA-MB-231, MCF-7, and GM12878. Not surprisingly, we identified all large deletions that were previously reported (See Additional file 7: Table S6 and Additional file 1: Figures S15 and S16) with their correct genotypes. Furthermore, PRINCESS was able to phase all (Additional file 1: Figure S19) but one SV across all samples. Only 1 heterozygous SV (in GM12878) was not phased due to the lack of heterozygote SNVs or indels in the region.

Lastly, we compared the methylation frequency results to the previous reports (see Additional file 8: Table S7). For the non-tumorigenic samples, we see high concordance (GM12878: 99.88% and MCF-10A: 99.02%). This is marginally reduced for the cancerous samples (MDA-MB-231: 98.80% and MCF-7: 98.52%). PRINCESS was able to phase the methylation data together with the SNVs, indels, and SVs revealing the entire biological picture of these regions across these samples (Additional file 1: Figure S19).

Again, PRINCESS shows a high concordance to previous studies. More importantly, it enables a fast and simple execution to more comprehensively study the sample at hand, even for a non-expert user.

### Analysis of a patient sample with Charcot-Marie-Tooth neuropathy

**Fig. 3:**
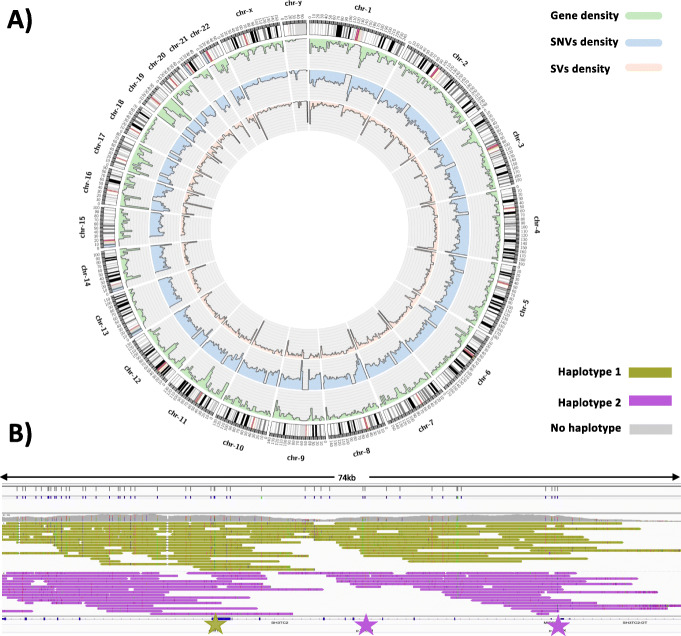
Overview of HS1011 analysis. **A** Circos plot showing the density of identified variants in a 4 Mbp window from outside in. Gene density (green), SNV and indel density (blue), SV density (reddish) for ONT sequence. **B** Phasing of SNVs, indels, and SVs across SH3TC2. PRINCESS was able to identify the three causative SNVs for Charcot-Marie-Tooth disease in this patient. The three stars indicate the location of the three variants and their individual haplotypes that they are assigned to

We applied PRINCESS to a human sample (HS1011) from a patient with Charcot-Marie-Tooth neuropathy (CMT), which was sequenced to ~ 18× coverage (read N50: 15,510bp) using ONT. This individual has been well characterized using multiple sequencing and genotyping platforms as reported previously [44, 45]. Figure 3A gives an overview of the identified variants across the entire genome.

Using PRINCESS, we identified 4,156,673 SNVs and indels (see Supplementary section S8). As expected, the majority (94.27%) of these are SNVs. For the smaller indels (1–50 bp), we observed a slight imbalance having a higher insertion number (419,614) than deletions (116,030). We further investigated the overlap with repeats and found that only 6.25% (259,886) of the SNVs and indels overlapped with simple repeats. The majority are again SNVs (80.96%), which highlights an improvement in the ONT technology with a balance between insertion (9.27%) and deletions (9.75%). Previous ONT basecalls had a strong bias in these regions for deletions [28].

As indicated above, this patient suffers from CMT disease that is an inherited genetic condition [44, 46, 47]. Previous studies highlighted the role of *SH3TC2* gene and mutations across it. Figure 3B shows the results for PRINCESS along *SH3TC2*. PRINCESS was able to identify multiple SNV and even SV (in intronic regions) and was further able to phase across the entire gene body. Three previous studies reported the mutations (*p.R954X*, *p.Y169H*, and *p.M1?*) for this patient, based on Illumina WES and WGS data [44–46]. Interestingly, only WES data with high coverage could identify *p.M1?* [46]. PRINCESS was able to identify all three of these mutations and, in addition, report that *p.Y169H* and *p.R954X* are on the same haplotype while *p.M1?* is on the opposite haplotype.

PRINCESS also identified 20,979 SVs across the entire genome. As expected, the majority of SVs are insertions (49.59%) followed by deletions (42.29%). We identified a higher concentration of SVs (63.64%) falling in simple repeats compared to SNVs and indels (6.25%) (see Additional file 9: Table S8). In contrast to previous reports [28], we found a more balanced distribution of SV type across these 6138 SVs: insertions (47.58%), deletions (45.97%), translocations (3.49%), duplications (2.00%), and inversions (0.94%). Interestingly, 79.72% of all the translocations overlap with repeats, with the majority over SINE and LINE elements (Additional file 10: Table S9). The slightly higher number of insertions than deletions is comparable to other studies [1] and thus indicates that the previous incorrect enrichment of deletions is not observed anymore [28]. Likewise, we identified a positive relationship between the identified SVs, SNVs, and indels per region (*R* = 0.35, *p* < 2.2e−16) (Additional file 1: Figure S12).

Using these SNV and SV variant calls, PRINCESS was able to phase 86.30% of the human genome with an N50 phase block of 821,907 bp. Overall, 92.17% of all heterozygous SNVs and indels were phased across 7059 phase blocks. Across these blocks, PRINCESS was able to phase only 30.41% of the heterozygous SV (similar to HG002). Here, PRINCESS detected at least a single read that is in conflict with the phasing information and decided to not phase the SV. This might also highlight general phasing issues of SNV around SV that might be amplified over the low coverage [42]. The phasing can be increased by allowing for some conflicting reads to be ignored as we showed over HG002 (see Fig. 2B).

**Fig. 4:**
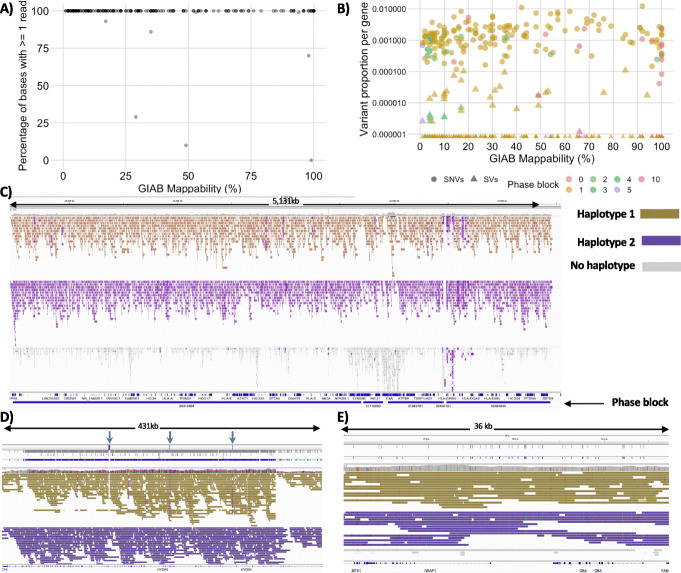
Overview across the 192 medically relevant, challenging genes. **A** Mappability vs coverage representation across the 192 hard to assess medical regions, highlighting the ability of long reads and PRINCESS to map the reads across these genes. **B** Variant calling (SNV: circles, SV: triangles) does not seem to be affected by the repetitiveness (GIAB Mappability) across these 192 regions. Furthermore, PRINCESS is able to successfully phase the majority of the genes indicated by color (number of phase blocks). **C** HLA gene showing phased reads across 5 phase blocks (bottom) over the full region. **D** PRINCESS is able to phase variants and reads across the entire HYDIN gene (431 kbp). **E** GBA and GBAP1 pseudogene in 36-kb regions showing both haplotypes aligned reads and the phased SNVs and indels

Lastly, we wanted to assess PRINCESS across medically relevant, but challenging regions. To achieve this, we used 193 medical genes that often escape a comprehensive analysis using NGS (Illumina) alone due to their repetitiveness [38] (see Additional file 11: Table S10 Additional file 1: Figure S13). Here we investigated how well PRINCESS could assess (i.e., mapping and variant calling) these genes (Fig. 4A–E) and how well these regions could be phased (see “Methods”). The average coverage across all genes was 17×, similar to the genome-wide coverage of 18×. Thus, highlighting a robust mapping and variant identification across the 192 genes across GRCh38 (Fig. 4A), as one gene could not have been lifted over (see “Methods”). When assessing the coverage per gene, we observed a few outliers either with very high coverage *DUX4* (111.98×) and *TCEB3c* (75.22×) or low/uncovered genes *CCL4L1* (0×) and *RHD* (0.33×).

Using PRINCESS, we identified 18,805 SNVs and indels and 100 SVs across the 189 (one gene did not show any variant, two genes had zero coverage, and one gene was not retrievable from Ensembl annotation) medically relevant genes, with an average of 101 SNVs and indels per gene. We identified 12,981 (69.02%) heterozygous SNVs and indels of which the majority 11,948 (92.04%) are phased (Fig. 3B). Overall, 90.35% of the 189 genes have one continuous phase block, with the highest outlier being *PCDH11X* (843,970 bp in length) having 10 phase blocks (see Fig. 4B and Additional file 1: Figure S14). Thus, PRINCESS was able to fully phase and resolve these hard-to-assess medically relevant genes (e.g., *LPA*, *GBA*).

The human leukocyte antigen (*HLA*) has been associated with numerous diseases, such as diabetes, rheumatoid arthritis, psoriasis, asthma, and various other autoimmune disorders [48]. *HLA* is a highly polymorphic ~ 4 Mbp region of the human genome and 3.85% of it are low mappability regions, which makes it typically hard to align (for short- and long reads alike [40]) and identify variants [9]. Likewise, it is an important genetic component of the immune system. We identified 21,102 variants, phased 95.02% of them in a total of 5 phase blocks across the entire ~ 4 Mbp *HLA* region. Figure 4C shows the results across IGV**.** Furthermore, we identified 116 SVs distributed as (63 deletions, 42 insertions, 3 duplications, 4 inversions, and 4 translocations.). *HYDIN* is a large (423 kb) gene, of which 75.56% are low mappability regions. For this hard to assess gene, we identified 2,041 SNVs and indels, most of which are heterozygous (97.69%). PRINCESS was able to phase 94.93% of the heterozygous SNVs and indels throughout the entire gene (Fig. 4D). Additionally, PRINCESS detected a deletion and two insertions in the introns of this gene (Fig. 4D marked with blue arrow). Lastly, glucocerebrosidase gene (*GBA*), which is 10,248 bp long and 26.05% of the gene is highly repetitive. Moreover, the presence of a highly homologous pseudogene (*GBAP1*) located downstream of the *GBA* gene can result in complex gene-pseudogene rearrangements, which makes the analysis of *GBA* challenging [49, 50]. Mutations in the *GBA* gene are responsible for Gaucher disease and represent the main genetic risk factor for developing Parkinson disease [51–53]. We identified 14 SNVs and indels and phased all of them in one phase block (Fig. 4E), thus, overall, highlighting the ability of PRINCESS to analyze medical regions that are otherwise difficult to comprehensively assess with even ~18× coverage ONT data.

## Discussion

Recent long-read studies often focus on either phasing of SNV, SV, or methylation detection [30, 31, 54]. Yet, the information of all three variant/modification types are available over long-read platforms [55]. Typically, one requires expert knowledge to accurately identify either of these variants (SNVs, indels, or SVs) or epigenetic changes using long-read technologies. Another often even bigger challenge is to fully leverage SNV, SV, and methylation information and combine them into one comprehensive picture of the sample at hand. For this purpose, we developed PRINCESS that shows a high accuracy (SNV 96.21–97.92%, SV 85.60–90.91%) across different sequencing applications and technologies. PRINCESS reports haplotype resolved SNV, SV, and methylation plus combines the results into two output files within an ~ 18-h runtime (Additional file 12: Table S11). Furthermore, as described in this paper, PRINCESS also achieves high accuracy in complex and repetitive regions across the genome.

PRINCESS itself is not only a workflow of existing tools, but rather includes multiple optimizations, QC approaches, and novel methods. Besides parameter optimizations, PRINCESS extends the principle of phasing variants to structural variations and also includes modules to phase methylation data (not shown here). This makes PRINCESS unique, as no other tool currently offers this level of comprehensiveness. Phasing SV, however, remains challenging as SV also often leads to problems for SNV calling and thus SNV phasing due to alignment artifacts or simple assumption violations, e.g., of heterozygote vs. homozygote ratios of SNV inside a duplication [42]. PRINCESS has by default conservative settings that do not phase a SV if one of the reads is showing a conflict. This leads to a lower phasing ability for HS1011. However, users can define a threshold to allow one or more reads to be in conflict to enable a higher phasing rate of SV itself. This in our experiments does not lead to a significantly higher Hamming error rate. In addition, PRINCESS includes code to enable the haplotype assessment of the methylation calls which provides a comprehensive foundation for maximal analysis of a given sample. In addition, PRINCESS has multiple optimization steps to fully utilize the methods incorporated. Therefore, PRINCESS adapts to the sample at hand and also runs across non-human or non-model organisms. The current version assumes the underlying genome is diploid for the reported genotypes and phasing approach. PRINCESS is highly configurable given a YAML parameter file along with command line parameters, and researchers can choose to use only subparts of PRINCESS. For example, if a specialized mapping is needed, one can provide the mapped reads (bam) to PRINCESS, and it will only proceed to call the variants. Furthermore, PRINCESS also accepts variant calls (e.g., SNV) from other technologies to leverage phasing. In the same manner, parental VCF files can be supplied to improve the phasing itself allowing for a better assessment of de novo variants.

While long-read sequencing remains expensive compared to short-read sequencing, it is clear that it has several advantages [1, 55]. The current LRS technologies continue to improve their sequencing yield, enabling a better cost model [1]. To further improve this cost model, PRINCESS is designed to also handle low coverage long-read data for both platforms. Given the current error rate in each platform, we recommend a minimum of 8× coverage for HiFi reads and > 12× for ONT sequencing data. As improvements continue with basecalling or the pore itself for ONT, we will see coverage requirements reduced in the near future. As an example, our sequencing run for HS1011 (~ 18× coverage) can be pooled with another sample on a single ONT flow cell, which further significantly reduces the price. The point can be made that a low coverage long-read sequencing run enables the assessment of more variants (e.g., insertions) and of certain regions (e.g., *HLA*) or other repeats compared to a 30–40× Illumina sequencing run. PRINCESS is capable of adapting to these different coverage levels and thus fully utilizes the long-read data at hand.

To demonstrate the utility of PRINCESS, we have benchmarked several whole genomes and targeted data sets using different read lengths and sequencing technologies. Using this same algorithm, we focused on 193 medically relevant genes that had been reported previously to cause issues when calling variants with short-reads alone [38]. These included well-known gene regions such as *HLA*, *LPA*, and others, but also Mendelian and neurological disease genes (e.g., *GBA*) [56–59]. We demonstrated that using a low coverage ONT run, we confidently identified variants across these genes as well as phased the majority of them to improve our insights for this particular sample. With only ~ 18× ONT coverage, we could phase through *HLA* resulting in only five phase blocks or fully phase and resolve *GBA* and its pseudogene (Fig. 4). The latter gene plays an important role in Parkinson’s disease or MSA. Since *GBA* is very repetitive, it leads to an ambiguous mapping for Illumina and so far is most often assessed with Sanger sequencing. PRINCESS could resolve this gene completely and will allow studying interesting recombination across *GBA* and *GBAP1*.

In summary, PRINCESS is a versatile method to obtain comprehensive insights into samples with long-read sequencing data and can also be used on low coverage datasets to enable a more detailed and complete foundation across the sample of interest.

## Methods

PRINCESS is implemented in Python (3.7.6) and uses Snakemake workflow [37] version 5.7.1 as its core. The framework is composed of different steps:

(1) Summary statistics for input sequence (Fasta or Fastq files could also be zipped). (2) Alignment using either Minimap2 [27] version 2.17-r941 or NGMLR [28] version 0.2.7. (3) (see below: “Alignment of long reads” section) Converting SAM to BAM and adding a read group (RG) field using samtools [60] (version 1.9). (4) Calling SNVs and indels using Clair [39] version 2.0.0 followed by filtering variants using PRINCESS tools, the SNVs, and indel quality values identified by Clair are distributed in two bell curved shape, to get the best results the threshold point between these two curves should be identified (based on https://github.com/HKU-BAL/Clair#pacbio-ccs-data). PRINCESS implements a filtering process that automatically identifies the SNV quality threshold, we compared the quality results for SNVs before and after using PRINCESS filter as well as the RTG benchmark for different technologies (HiFi, CLR, and ONT), more in section 2.1.1 supplementary material. (5) Call SVs using Sniffles [28] version 1.0.12. (6) Phasing identified SNVs and indels using WhatsHap phase [32] version 0.18 (see below: Phasing of SNV, indel, SV and methylation in a harmonized way). (7) Haplotype the BAM file using WhatsHap haplotag. (8) Split the haplotyped reads from the BAM file based on the phase block and haplotype value using PRINCESS tools. (9) Phasing SVs using PRINCESS tools and information retrieved from step 7. Optional steps: (10) PRINCESS can improve the phasing using the parental SNPs. Here we use BCFtools [61] version 1.9 to merge parental SNPs with the identified SNVs and indels from PRINCESS and later use PRINCESS tools to update the SNVs and indels haplotype. (11) PRINCESS also identifies methylation events using Nanopolish [62] (version 0.11.2.) and tries to phase the methylation information based on SNV phasing using PRINCESS tools**.**

### Alignment of long reads

We use Minimap2 as the default aligner, but the user can change that using -a parameter to choose NGMLR instead (more accurate but slower). Based on the read type specified by the user, PRINCESS will implicitly choose the optimum aligning parameters. For Minimap2, if the input reads are PacBio, we use the default parameters, plus -H, -a, and -x map-pb to specify use of homopolymer-compressed *k-mer*, we use samtools to convert to a BAM file. By default, we use five threads, which can be changed from the config file. But, if the input reads are ONT, the default parameters will be used, and -x will be set to map-ont instead. In both cases (PacBio or ONT),we use the --MD flag to add MD to the aligned output. In NGMLR, we use the flag -x to identify read type either PacBio or ONT and --bam-fix to report reads with > 64 k CIGAR operations as unmapped. As we did in Minimap2, samtools is used here to convert and index the output SAM file.

Note: if there is more than one input file (PacBio or ONT), each file will be aligned separately then merged using samtools merge (if the user is using a cluster, each job will run on a separate node).

### Identifying genomic variations and alterations

We start from the previous step, where we have a sorted and indexed bam file containing the RG and MD flag. We use Clair2 to identify SNVs and indels. Clair2 uses a deep neural network to detect variants, and based on the sequence read type (HiFi, CLR, or ONT), we choose the adequate model. Likewise, we speed the process of calling variants by two steps. First, calling each chromosome separately rather than calling all the datasets at once. Second, splitting the chromosomes into equal regions, with a minimum length of 24,925,062 bp (this is the optimum value, the user can change this value by changing the chr_split field in the config file). We use the callVarBam algorithm with five threads and minimum read support of 2 to identify SNVs and indels. The identified SNVs and indels are implicitly filtered using PRINCESS sub-tools (the user can ignore this step using the -t option). Lastly, all the identified regions per chromosome will be merged, sorted, and tabix, using vcfcat and vcfstreamsort.

To call SVs, we use Sniffles 1.0.12, which takes a BAM indexed file as input. We use a minimum of 3 reads to support SV (users can change this behavior by changing the sniffles_coverage field in the config file.)

Methylation is an optional step in PRINCESS. To activate this process, the user needs to use the -m flag and support the fast5 directory using -md (-m and -md parameters are mutually inclusive). PRINCESS detects methylation using ONT data. First, each Fasta/Fastq file is indexed using the Nanopolish index. Later, these files are used with bam files to call methylation using Nanopolish call methylation with default parameters using eight threads, which is changeable from config file field methylation_threads.

### Phasing of SNV, indel, SV, and methylation in a harmonized way

Identified SNVs and indels phased using the WhastHap phase default algorithm (whatshap). To run phasing, PRINCESS uses the BAM file plus the identified SNVs and indels. Default parameters are used to phase variants, Read Groups (RG) are ignored, and the sequence reads used for phasing are printed to a file in the same directory as the phased SNVs and indels, with the “.reads” extension. To phase SVs, first, we use the phased SNVs and indels plus the BAM file to haplotype reads in the BAM. We achieve that by using WhatsHap haplotag with default parameters. Only reads with tag information are selected from the BAM file in addition to its haplotype and phase block information. PRINCESS sub-tools use this information to produce a new VCF file with two extra fields. PS field, which indicates the phasing block, CONFLICT field, which gives information if there is a conflict between the sequence reads while identifying the PS value. Lastly, after detecting methylations, read information from the tagged file is used to add phasing information (PS) and haplotype (HP) fields for each methylation group using PRINCESS sub-tools. The resulting file is tab-delimited, which contains methylation group information and read supporting that, beside PS and HP tag. If the information is not available for this read, we substitute it with “.”.

It is possible for PRINCESS to utilize parental SNPs to leverage phasing of the identified SNVs and indels, as well as reducing false-positive results. First, we merge the identified SNVs and indels with paternal and maternal SNPs, respectively. Later, this file is used by PRINCESS sub-tools to produce a new VCF file with high confidence phased and haplotype SNPs. We use a tolerance ratio of 5% and a minimum of 10 SNPs per block to be identified as rightly phased.

### Configurations

PRINCESS uses a YAML file for configuration. Additionally, the Python wrapper we developed around Snakemake accepts inputs from the user to override the current parameter used in the configuration file.

### Variant benchmarking

PRINCESS was benchmarked using PacBio and ONT publicly available data for HG002 from GIAB [40] based on GRCh37 (hg19). We benchmarked SNV and indel calling using RTG [63] version 3.9.1 and available GIAB SNV data version 3.3.2 [40]. For SV, we used the version 0.6 benchmark set from GIAB and Truvari version 0.1.0 (https://github.com/spiralgenetics/truvari) with recommended parameters. We benchmarked phasing (switch error and Hamming error rate) using WhatsHap “compare” and calculated N50 using WhatsHap “stats”. The data was down-sampled using samtools view to 5×, 10×, and 25× coverage for ONT and HiFi data and 5×, 10×, 25×, and 50× for CLR and evaluated the results from PRINCESS.

### Phasing benchmark

For the phasing benchmark, we used both WhatsHap algorithms stat and compare together with data from GIAB gold standard to calculate Hamming and switch error rate, likewise, N50.

### Cas9 targeted assay comparison

Capture data from Gilpatrick et al. [43] were aligned and compared to GRCh38 (the previous study was done using GRCh38). Likewise, we benchmarked SNVs and indels using bcftools merge, and we intersected our identified methylation with reported results using bedtools intersect [64] version 0.2.6. Lastly, for the SVs, we compared the breakpoint reported from Gilpatrick et al. and both SV type and genotype to what PRINCESS reported.

### Long-read sequencing of HS1011

DNA was sheared to 30 kb using a Diagnode Megarupter following the manufacturer’s recommendations. DNA was prepared for Nanopore sequencing using the ONT 1D sequencing by ligation kit (SQK-LSK109). Briefly, 1–1.5 μg of fragmented DNA was end-repaired with the NEB FFPE repair kit, followed by end repair and A-tailing with the NEB Ultra II end-prep kit. After an Ampure clean up step, prepared fragments were ligated to ONT-specific adapters via the NEB blunt/TA master mix kit. The library underwent a final clean up and was loaded onto a PromethION PRO0001 flow cell per the manufacturer’s instructions. The flow cell was sequenced with standard parameters for 3 days. Basecalling was carried out via Guppy version 4.3.4 + ecb2805. The sample was analyzed using PRINCESS and the GRCh37 reference. We reported the number of SNVs, indels, and SV density in 4 Mbp using vcftools [65] version 0.1.13.

### Medical gene benchmarking

We measure the performance of PRINCESS across 193 difficult to map medical genes [38]. The coordinates for these regions were extracted from the GTF file for genome 37. The medical regions were intersected using bedtools [64] with a low mappability track (ftp://ftp-trace.ncbi.nlm.nih.gov/ReferenceSamples/giab/release/genome-stratifications/v2.0/GRCh37/mappability/) to identify the percentage of repeats base pairs for each gene. We used mosdepth [66] version 0.2.6 with bedtools intersect to calculate average coverage and zero covered bases in each gene. We identified the intersected SNVs and indels and SVs breakpoint with these regions using bedtools.

## Supplementary Information

**Additional file 1.** Supplementary material and figures.

**Additional file 2: Table S1.** PacBio HiFi data with different insert size SNVs and indels calling benchmark with and without filtering.

**Additional file 3: Table S2.** Table of SNVs and indels benchmarking using PRINCESS filter.

**Additional file 4: Table S3.** Genotype benchmarking count across different algorithms, Clair2, WhatsHap genotype, WhatsHap trust, and WhatsHap untrust.

**Additional file 5: Table S4.** Structural variant benchmarking.

**Additional file 6: Table S5.** Phasing benchmarking.

**Additional file 7: Table S6.** Capture data SV benchmark.

**Additional file 8: Table S7.** Capture data methylation benchmark.

**Additional file 9: Table S8.** Sample HS1011 variant intersection with repeat regions.

**Additional file 10: Table S9.** Count of transposons repeat intersecting with the medical relevant genes.

**Additional file 11: Table S10.** Repeat percentage in 193 medical relevant genes.

**Additional file 12: Table S11.** Run time for PacBio HiFi data.

**Additional file 13: Table S12.** Data availability.

**Additional file 14: Table S13.** Summary of the identified variant in the sample HS1011.

**Additional file 15: Table S14.** Genotype benchmark percentage using different algorithms.

**Additional file 16: Table S15.** Structural variant phasing benchmark.

**Additional file 17: Table S16.** PacBio HiFi different insert size SV benchmark.

**Additional file 18: Table S17.** PacBio HiFi different insert size SNVs and indels benchmark.

**Additional file 19: Table S18.** PacBio HiFi with different insert size phasing benchmark.

**Additional file 20: Table S19.** SNVs and indels benchmarking without PRINCESS filter.

**Additional file 21.** Review history.

## Data Availability

CLR data available at ftp://ftp-trace.ncbi.nlm.nih.gov/ReferenceSamples/giab/data/AshkenazimTrio/HG002_NA24385_son/PacBio_MtSinai_NIST/PacBio_fasta/, HiFi data ftp://ftp-trace.ncbi.nlm.nih.gov/ReferenceSamples/giab/data/AshkenazimTrio/HG002_NA24385_son/PacBio_CCS_15kb/, and ONT ftp://ftp-trace.ncbi.nlm.nih.gov/ReferenceSamples/giab/data/AshkenazimTrio/HG002_NA24385_son/Ultralong_OxfordNanopore/ Sample HS1011 [67] is submitted to SRA under bioproject PRJNA203659 Capture data [43] BioProject ID PRJNA531320. Additional file 13: Table S12 shows all the data sets that were used for this paper. PRINCESS source code [68], documentation, and manual are available at https://github.com/MeHelmy/princess under the MIT license code 10.5281/zenodo.5272109.

## References

[1] Coster WD, De Coster W, Weissensteiner MH, Sedlazeck FJ. Towards population-scale long-read sequencing [Internet]. Nat Rev Genet. 2021; Available from: 10.1038/s41576-021-00367-3. 10.1038/s41576-021-00367-3 PMC8161719 34050336

[2] Logsdon GA, Vollger MR, Eichler EE (2020). Long-read human genome sequencing and its applications. Nat Rev Genet..

[3] Wenger AM, Peluso P, Rowell WJ, Chang P-C, Hall RJ, Concepcion GT (2019). Accurate circular consensus long-read sequencing improves variant detection and assembly of a human genome. Nat Biotechnol..

[4] Logsdon GA, Vollger MR, Hsieh P, Mao Y, Liskovykh MA, Koren S, et al. The structure, function and evolution of a complete human chromosome 8. Nature. 2021;593:101–7. 10.1038/s41586-021-03420-7 10.1038/s41586-021-03420-7 PMC8099727 33828295

[5] Miga KH, Koren S, Rhie A, Vollger MR, Gershman A, Bzikadze A (2020). Telomere-to-telomere assembly of a complete human X chromosome. Nature.

[6] Alonge M, Wang X, Benoit M, Soyk S, Pereira L, Zhang L (2020). Major impacts of widespread structural variation on gene expression and crop improvement in tomato. Cell.

[7] Beyter D, Ingimundardottir H, Oddsson A, Eggertsson HP, Bjornsson E, Jonsson H (2021). Long-read sequencing of 3,622 Icelanders provides insight into the role of structural variants in human diseases and other traits. Nat Genet..

[8] Chen X, Sanchis-Juan A, French CE, Connell AJ, Delon I, Kingsbury Z (2020). Spinal muscular atrophy diagnosis and carrier screening from genome sequencing data. Genet Med..

[9] Chin C-S, Wagner J, Zeng Q, Garrison E, Garg S, Fungtammasan A (2020). A diploid assembly-based benchmark for variants in the major histocompatibility complex. Nat Commun..

[10] Wagner J, Olson ND, Harris L, et al. Towards a comprehensive variation benchmark for challenging medically-relevant autosomal genes. bioRxiv; 2021. 10.1101/2021.06.07.444885.

[11] Aganezov S, Goodwin S, Sherman RM, Sedlazeck FJ, Arun G, Bhatia S (2020). Comprehensive analysis of structural variants in breast cancer genomes using single-molecule sequencing. Genome Res..

[12] Mahmoud M, Gobet N, Cruz-Dávalos DI, Mounier N, Dessimoz C, Sedlazeck FJ. Structural variant calling: the long and the short of it [Internet]. Genome Biol. 2019; Available from: 10.1186/s13059-019-1828-7. 10.1186/s13059-019-1828-7 PMC6868818 31747936

[13] Ho SS, Urban AE, Mills RE (2020). Structural variation in the sequencing era. Nat Rev Genet..

[14] Weissensteiner MH, Bunikis I, Catalán A, Francoijs K-J, Knief U, Heim W, et al. Discovery and population genomics of structural variation in a songbird genus [Internet]. Nat Commun. 2020; Available from: https://doi.org/10.1038/s41467-020-17195-4. 10.1038/s41467-020-17195-4 PMC7341801 32636372

[15] Pollard MO, Gurdasani D, Mentzer AJ, Porter T, Sandhu MS (2018). Long reads: their purpose and place. Hum Mol Genet..

[16] Alekseyev YO, Fazeli R, Yang S, Basran R, Maher T, Miller NS, et al. A next-generation sequencing primer—how does it work and what can it do? Acad Pathol. 2018:237428951876652 Available from: 10.1177/2374289518766521. 10.1177/2374289518766521 PMC5944141 29761157

[17] Inc. KN, Kernel Networks Inc. Mitochondrial Diseases - Long-read Genome and Transcriptome Sequencing in Cases Unresolved After Short-read Genomics [Internet]. Case Med Res. 2019; Available from: 10.31525/ct1-nct03962452.

[18] Murdock D, Rosenfeld J, Xia F, Burrage L, Mahmoud M, Sedlazeck F, et al. Long-read sequencing for diagnosis in the Undiagnosed Diseases Network [Internet]. Mol Genet Metab. 2021:S253–4 Available from: 10.1016/s1096-7192(21)00471-6.

[19] Tusso S, Nieuwenhuis BPS, Sedlazeck FJ, Davey JW, Jeffares DC, Wolf JBW (2019). Ancestral admixture is the main determinant of global biodiversity in fission yeast. Mol Biol Evol..

[20] Frazer KA, Murray SS, Schork NJ, Topol EJ. Human genetic variation and its contribution to complex traits [Internet]. Nat Rev Genet. 2009:241–51 Available from: 10.1038/nrg2554. 10.1038/nrg2554 19293820

[21] Kilpinen H, Dermitzakis ET. Genetic and epigenetic contribution to complex traits [Internet]. Hum Mol Genet. 2012:R24–8 Available from: 10.1093/hmg/dds383. 10.1093/hmg/dds383 22976472

[22] Hirschhorn JN, on Behalf of the Genetic Investigation of Anthropometric Traits (GIANT) Consortium. The identification of 180 genetic loci involved in adult height variation highlights biological pathways and provides insights into the contribution of common genetic variation to human growth [Internet]. The Endocrine Society’s 92nd Annual Meeting, June 19–22, 2010 - San Diego. 2010. p. OR43–4. Available from: 10.1210/endo-meetings.2010.part3.or2.or43-4.

[23] Carvalho CMB, Ramocki MB, Pehlivan D, Franco LM, Gonzaga-Jauregui C, Fang P (2011). Inverted genomic segments and complex triplication rearrangements are mediated by inverted repeats in the human genome. Nat Genet..

[24] Weischenfeldt J, Symmons O, Spitz F, Korbel JO (2013). Phenotypic impact of genomic structural variation: insights from and for human disease. Nat Rev Genet..

[25] Cheng H, Concepcion GT, Feng X, Zhang H, Li H (2021). Haplotype-resolved de novo assembly using phased assembly graphs with hifiasm. Nat Methods..

[26] Shafin K, Pesout T, Lorig-Roach R, Haukness M, Olsen HE, Bosworth C (2020). Nanopore sequencing and the Shasta toolkit enable efficient de novo assembly of eleven human genomes. Nat Biotechnol.

[27] Li H (2018). Minimap2: pairwise alignment for nucleotide sequences. Bioinformatics..

[28] Sedlazeck FJ, Rescheneder P, Smolka M, Fang H, Nattestad M, von Haeseler A (2018). Accurate detection of complex structural variations using single-molecule sequencing. Nat Methods..

[29] Luo R, Sedlazeck FJ, Lam T-W, Schatz MC (2019). A multi-task convolutional deep neural network for variant calling in single molecule sequencing. Nat Commun.

[30] Edge P, Bansal V (2019). Longshot enables accurate variant calling in diploid genomes from single-molecule long read sequencing. Nat Commun..

[31] Jiang T, Liu Y, Jiang Y, Li J, Gao Y, Cui Z (2020). Long-read-based human genomic structural variation detection with cuteSV. Genome Biol..

[32] Patterson M, Marschall T, Pisanti N, van Iersel L, Stougie L, Klau GW (2015). WhatsHap: weighted haplotype assembly for future-generation sequencing reads. J Comput Biol..

[33] Simpson JT, Workman RE, Zuzarte PC, David M, Dursi LJ, Timp W (2017). Detecting DNA cytosine methylation using nanopore sequencing. Nat Methods.

[34] Liu Q, Fang L, Yu G, Wang D, Xiao C-L, Wang K (2019). Detection of DNA base modifications by deep recurrent neural network on Oxford Nanopore sequencing data. Nat Commun..

[35] De Coster W, D’Hert S, Schultz DT, Cruts M, Van Broeckhoven C (2018). NanoPack: visualizing and processing long-read sequencing data. Bioinformatics.

[36] Zhang H, Jain C, Aluru S (2020). A comprehensive evaluation of long read error correction methods. BMC Genomics.

[37] Köster J, Rahmann S. Snakemake—a scalable bioinformatics workflow engine [Internet]. Bioinformatics. 2018:3600–0 Available from: 10.1093/bioinformatics/bty350. 10.1093/bioinformatics/bty350 29788404

[38] Mandelker D, Schmidt RJ, Ankala A, Gibson KM, Bowser M, Sharma H, et al. Navigating highly homologous genes in a molecular diagnostic setting: a resource for clinical next-generation sequencing [Internet]. Genet Med. 2016:1282–9 Available from: 10.1038/gim.2016.58. 10.1038/gim.2016.58 27228465

[39] Luo R, Wong CL, Wong YS, et al. Clair: Exploring the limit of using a deep neural network on pileup data for germline variant calling. Available from: 10.1101/865782.

[40] Zook JM, McDaniel J, Olson ND, Wagner J, Parikh H, Heaton H (2019). An open resource for accurately benchmarking small variant and reference calls. Nat Biotechnol..

[41] Zook JM, Hansen NF, Olson ND, Chapman L, Mullikin JC, Xiao C (2020). A robust benchmark for detection of germline large deletions and insertions. Nat Biotechnol..

[42] Wagner J, Olson ND, Harris L, et al. Benchmarking challenging small variants with linked and long reads. bioRxiv; 2021. Available from: 10.1101/2020.07.24.212712. 10.1016/j.xgen.2022.100128 PMC9706577 36452119

[43] Gilpatrick T, Lee I, Graham JE, Raimondeau E, Bowen R, Heron A (2020). Targeted nanopore sequencing with Cas9-guided adapter ligation. Nat Biotechnol..

[44] Lupski JR, Reid JG, Gonzaga-Jauregui C, Rio Deiros D, Chen DCY, Nazareth L (2010). Whole-genome sequencing in a patient with Charcot-Marie-Tooth neuropathy. N Engl J Med..

[45] English AC, Salerno WJ, Hampton OA, Gonzaga-Jauregui C, Ambreth S, Ritter DI (2015). Assessing structural variation in a personal genome-towards a human reference diploid genome. BMC Genomics..

[46] Lupski JR, Gonzaga-Jauregui C, Yang Y, Bainbridge MN, Jhangiani S, Buhay CJ (2013). Exome sequencing resolves apparent incidental findings and reveals further complexity of SH3TC2 variant alleles causing Charcot-Marie-Tooth neuropathy. Genome Med..

[47] English AC, Salerno WJ, Reid JG (2014). PBHoney: identifying genomic variants via long-read discordance and interrupted mapping. BMC Bioinformatics..

[48] Shiina T, Hosomichi K, Inoko H, Kulski JK (2009). The HLA genomic loci map: expression, interaction, diversity and disease. J Hum Genet..

[49] Zampieri S, Cattarossi S, Bembi B, Dardis A (2017). GBA analysis in next-generation era: pitfalls, challenges, and possible solutions. J Mol Diagn..

[50] Straniero L, Rimoldi V, Samarani M, Goldwurm S, Di Fonzo A, Krüger R (2017). The GBAP1 pseudogene acts as a ceRNA for the glucocerebrosidase gene GBA by sponging miR-22-3p. Sci Rep..

[51] Yu Z, Wang T, Xu J, Wang W, Wang G, Chen C (2015). Mutations in the glucocerebrosidase gene are responsible for Chinese patients with Parkinson’s disease. J Hum Genet..

[52] Aslam M, Kandasamy N, Ullah A, Paramasivam N, Öztürk MA, Naureen S (2021). Putative second hit rare genetic variants in families with seemingly GBA-associated Parkinson’s disease. NPJ Genom Med..

[53] Weber M, Min S-W, Truong T, Hung J, Dale S, Reichelt M (2021). Ocular phenotypes in a mouse model of impaired glucocerebrosidase activity. Sci Rep.

[54] Roberts HE, Lopopolo M, Pagnamenta AT, Sharma E, Parkes D, Lonie L (2021). Short and long-read genome sequencing methodologies for somatic variant detection; genomic analysis of a patient with diffuse large B-cell lymphoma. Sci Rep.

[55] Sedlazeck FJ, Lee H, Darby CA, Schatz MC (2018). Piercing the dark matter: bioinformatics of long-range sequencing and mapping. Nat Rev Genet..

[56] Riboldi GM, Di Fonzo AB. Gaucher disease, and Parkinson’s disease: from genetic to clinic to new therapeutic approaches. Cells. 2019;8 Available from: 10.3390/cells8040364. 10.3390/cells8040364 PMC6523296 31010158

[57] Sidransky E, Lopez G (2012). The link between the GBA gene and parkinsonism. Lancet Neurol..

[58] Burgess S, Ference BA, Staley JR, Freitag DF, Mason AM, Nielsen SF (2018). Association of LPA variants with risk of coronary disease and the implications for lipoprotein(a)-lowering therapies: a Mendelian randomization analysis. JAMA Cardiol..

[59] D’Antonio M, Reyna J, Jakubosky D, Donovan MK, Bonder M-J, Matsui H, et al. Systematic genetic analysis of the MHC region reveals mechanistic underpinnings of HLA type associations with disease. Elife. 2019;8 Available from: 10.7554/eLife.48476. 10.7554/eLife.48476 PMC6904215 31746734

[60] Li H, Handsaker B, Wysoker A, Fennell T, Ruan J, Homer N (2009). The Sequence Alignment/Map format and SAMtools. Bioinformatics..

[61] Li H (2011). A statistical framework for SNP calling, mutation discovery, association mapping and population genetical parameter estimation from sequencing data. Bioinformatics..

[62] Loman NJ, Quick J, Simpson JT (2015). A complete bacterial genome assembled de novo using only nanopore sequencing data. Nat Methods..

[63] Cleary JG, Braithwaite R, Gaastra K, Hilbush BS, Inglis S, Irvine SA, et al. Comparing variant call files for performance benchmarking of next-generation sequencing variant calling pipelines [Internet]. Cold Spring Harbor Lab. 2015:023754 [cited 2021 Feb 25]. Available from: https://www.biorxiv.org/content/10.1101/023754v2.abstract.

[64] Quinlan AR, Hall IM (2010). BEDTools: a flexible suite of utilities for comparing genomic features. Bioinformatics..

[65] Danecek P, Auton A, Abecasis G, Albers CA, Banks E, DePristo MA (2011). The variant call format and VCFtools. Bioinformatics..

[66] Pedersen BS, Quinlan AR (2018). Mosdepth: quick coverage calculation for genomes and exomes. Bioinformatics..

[67] Mahmoud M, Sedlazeck F. Available from: “Genomic Sequencing of a Personal Human Genome Using Multiple next-Generation Sequencing Technologies.” n.d. https://www.ncbi.nlm.nih.gov/bioproject/PRJNA203659.

[68] Mahmoud M, Sedlazeck F. MeHelmy/princess: v1.0 [Internet]. Zenodo; 2021. Available from: https://zenodo.org/record/5272109.

