# Supplementary material for: PRINCESS: comprehensive detection of haplotype resolved SNVs, SVs, and methylation
Source: Genome Biol. 2021 Sep 14;22:268. doi: 10.1186/s13059-021-02486-w (PMC8442460; doi:10.1186/s13059-021-02486-w)
Supplement: Supplementary file 1 — Additional file 1. Supplementary material and figures. [file 13059_2021_2486_MOESM1_ESM.docx]

PRINCESS: comprehensive detection of haplotype resolved SNVs, SVs, and methylation.

Supplementary Materials

[**Benchmarking**](#_a7j6fgu0gjxu) **1**

[**Single Nucleotide Variants and indels**](#_5hx73oj5zw6i) **4**

[Filtering](#_f3dagr7gxduf) 4

[Compare SNVs and indels between different insert sizes](#_tjfuft9aj4s5) 4

[**Genotype**](#_fa24oedbkx2q) **5**

[**Structural Variants**](#_fa24oedbkx2q) **5**

[**Phasing**](#_fa24oedbkx2q) **6**

[**Methylation**](#_fa24oedbkx2q) **6**

[**Datasets**](#_wgyvp1ixu2l) **6**

[**HS1011**](#_o3nxy2637ap) **6**

[**Figures**](#_rlvcd3k4sm3) **7**

# Benchmarking

**Supplementary Fig: S1-3** show the data set properties. First, we are going to discuss the performance of SNVs and indels calling based on v.3.3.2 of the GIAB SNV and indels calls (see Methods for details). **Figure 1B** shows the results for F-measure (i.e. harmonic mean of precision and sensitivity) over different coverage levels for each of the sequencing technologies. PRINCESS employs its own filtering strategy for SNVs (**Supplementary Fig S7-11)**, which is described and benchmarked in **Supplementary Section 1.1.1**. **Supplementary Table S2** and **S4** show the detailed results across different coverage levels for SNV and SV calling, respectively. Overall sequencing technologies, PRINCESS showed a high level of performance (F-measure) for HiFi PacBio (97.60%) followed by CLR PacBio (94.06%) and ONT (91.85%) and achieved over 99% genotype accuracy (**Supplementary section 1.2, Supplementary Table s13 Fig S4**).

PRINCESS was specially tuned to obtain high precision across PacBio HiFi (97.92%), PacBio CLR (99.03%), and ONT (96.21%). Thus, the F-score is mainly impacted by the sensitivity observed for PacBio HiFi (97.28%), PacBio CLR (89.57%), and ONT (84.90%). The first results are based on the full data sets for PacBio HiFi (32x aligned coverage), PacBio CLR (95x aligned coverage), and ONT (53x aligned coverage). Next, we investigated the impact of indels on the performance, since these are the primary errors from the long-read sequencing technologies. Hence, when considering single mutations only the F-measure improved for PacBio HiFi (99.53%) followed by PacBio CLR (98.73%) and ONT (97.20%) with an average precision of 99.03% across the technologies.

As the coverage levels are hard to compare to each other, we benchmarked PRINCESS on 25x coverage across the sequencing technologies. Maybe not surprisingly, we still observed the highest F-measure for PacBio HiFi (97.11%), but now followed by ONT (84.45%) and PacBio CLR (82.29%). That is impacted by the overall precision of PRINCESS shown by HiFi (97.27%), CLR (96.29%), and ONT (90.87%). For SNVs only, we observed HiFi (99.46%) with the highest F-measure followed by CLR (97.00%) and ONT (89.70%). The reduced performance of ONT data is likely due to the inclusion of the ultra-long data in the read set, as well as our current SNV and indel filter working better on PacBio data. Next, we compare the performance across the technologies with adopted coverage values HiFi (10x), CLR (25x), and ONT (25x) to better represent a typical long-read sequencing run. For these coverage levels, we observed an F-measure for HiFi (78.59%) to be actually below the levels of ONT (84.45%) and PacBio CLR (82.29%). The reduction in HiFi is mainly driven by the drop in sensitivity (65.08%) whereas the precision (99.16%) remains high. For SNVs, HiFi (82.41%) shows a slightly reduced F-measure but still have the a highest precision (99.53%). Overall, PRINCESS shows a high accuracy for SNV and indel calling across all sequencing platforms.

Also, we benchmark PRINCESS for the detection of SVs across the same sequencing technologies (see methods). **Figure 1B** shows the results for F-measure over different coverage levels for each of the sequencing technologies. For the total coverage data sets, we observe the highest F-measure for PacBio HiFi (92.48%) followed by CLR (89.90%) and ONT (86.90%). This is influenced by the overall high precision for HiFi (90.91%) followed by CLR (90.00%) and ONT (88.50%). This performance drops marginally when considering only 25x for each of the technologies. We observed F-measures for each HiFi (92.00%), PacBio CLR (84.90%), and ONT (82.28%) with a slight increase in precision across all data sets (see **Supplementary Table S4**).

Lastly, looking at coverage of (HiFi: 10x, ONT, CLR: 25x), the result also change slightly. For HiFi (86.00%), we observed only a reduction of 0.06% in F-measure and thus make it very compatible with CLR and ONT.

PRINCESS not only identifies SNVs, indels, and SVs based on one dataset but additionally aims to phase the variations to provide a more comprehensive insight into the sample. We evaluated the SNVs and indels phasing, using WhatsHap and the truth dataset, where we measured switch error (i.e. multiple SNVs assigned to the wrong haplotype) and Hamming error (i.e. total number of wrongly assigned SNVs to haplotypes). We found that: the longest N50 is achieved by ONT (17,427kbp) followed by CLR (151kbp), and HiFi (117kbp) respectively. Although ONT achieved the highest N50, it suffered from a high Hamming error rate (0.19) **(see Figure 2A)**, but with a switch error rate of 0.0036 similar to CLR data, which achieved a lower Hamming error of (0.01032). This highlights smaller inconsistencies (Hamming error) compared to large phase block errors (switch error rates). Lastly, HiFi data achieved the lowest switch (0.0040) and Hamming (0.0052) error rates (**Supplementary Fig S5**). Overall the three technologies have very low switch error rates, but the longest N50 achieved by using ONT (**Supplementary Fig S6**) associated with a higher Hamming error rate than both HiFi and CLR data. Furthermore, we compared the performance of the three technologies using different coverage (**Supplementary Table S5**).

Additionally, we assessed the performance of PRINCESS to phase SVs. Interestingly, we observed the highest phase rate (77.17%) from HiFi followed by ONT (38.24%) and CLR (21.44%) (See **Supplementary Table S14**). This might be due to that we identified single reads conflicting with the phasing information for ONT or CLR, and thus PRINCESS does not assign the SV to the individual phase block. This can be adjusted by the user if needed.

Lastly, we wanted to measure the impact of the different available HiFi read lengths/insert sizes (15, 19, 20, and 25 Kb) on the ability to detect variations and phase them using PRINCESS. We analyzed HiFi data with different insert sizes. **Supplementary Fig S18B** shows the comparison between insert size for precision, recall, and F-measure (**see Supplementary Table S15 and S16**). Overall we observed a high F-measure for SNVs and indels calling using PRINCESS across the different insert sizes: 15kb (97.60%), 20kbp (97.65%), and even if the coverage is below 17x for 19kb (91.20%) and 25kbp (91.80%) (**see Supplementary section S1.1.2**). We noted a small reduction in recall/sensitivity for larger insert sizes, which we hypothesize is due to the lower number of cycles and thus higher consensus level errors for the longer HiFi reads. **Supplementary Fig S18A** illustrates the results for SV showing also similar high F-measurements using PRINCESS across 15kb (92.48%), 19kb (89.24%), 20kb (89.67%), and 25kb (89.34%). Thus, showing that the insert size and coverage only marginally affect the performance of PRINCESS. Lastly, we investigated the consequence of the insert sizes on phasing SNVs and SVs. **Supplementary** **Fig S18C and S18D and Supplementary Table S17** show the Hamming error rate and N50 phasing performance. As expected the N50 increases constantly with an increase in insert size from 15kb (N50: 117kb, Hamming error: 0.00521), 19kb (N50: 194kb, Hamming error: 0.01477), 20kb (N50: 201kb, Hamming error: 0.00869), to 25kb (N50: 257kb, Hamming error: 0.01415).

## Single Nucleotide Variants and indels

### Filtering

Quality values of the identified SNVs and indels by Clair2 are distributed in two bell-curved shapes as shown in **Supplementary** **Fig S7-9,** to get the optimum result, the threshold is the lowest point between these two curves (based on https://github.com/HKU-BAL/Clair2#pacbio-ccs-data), which is affected by sequence read type as well as coverage. PRINCESS implements a filtering process that automatically identifies SNVs and indels quality thresholds. We compared the quality results for SNVs and indels before and after using the PRINCESS filter likewise the RTG benchmark for different technologies (HiFi, CLR, and ONT) **Supplementary Fig S7-9, Supplementary Table S18** shows the precision, recall, and F1-score before filtering. We can see the filtering process implemented by Princes achieved a good result, **Supplementary Table S2** and increased F1 measure for identified SNVs and indels from all technologies.

### Compare SNVs and indels between different insert sizes

We used different HiFi insert sizes 15, 19, 20, and 25 Kb, the raw read coverage is ~29, ~15, ~17, and ~11x, and their N50 13,480, 17,988, 18,345, 22,610 respectively. We identified on average 4 million SNVs for insert size 15kb (4,504,207), 19kb (5,618,467), 20kb (4,461,158), and 25kb (3,947,024).

Likewise, we investigated the performance across each type of variant (SNVs, insertions, and deletions), as in the case of indels (insertions and deletions) the size of the variant may affect the benchmarking. For SNVs, we identified high F1 across the different insert sizes, 15kb (99.53%), 19kb (98.46%), 20kb (99.01%), and 25kb (94.32%), we can see that coverage affected the F1 measure especially when we look at the sample with insert size 19 and 25Kb where the coverage decreased from ~17x in 19 kb sample to ~11 in 25Kb sample the F1 measure dropped from 98.46% to 94.32%, besides, we can see increasing the insert-size leads to a small drop in the recall. For detailed information and benchmark using different coverage for each insert size (**Supplementary Table S16-17**)

## Genotype

To optimize the identified genotype (GT), we compared the initially identified GT by Clair2 with the gold standard from GIAB using bcftools merge. Later, we re-genotype the SNVs and indels using the WhatsHap genotype option to see if that will enhance the identified GT by Clair2, followed by comparing it as the immediate step with the GIAB gold standard. Additionally, in our workflow, we use WhatsHap to phase the detected SNVs and indels, which have two options: either trust the GT or distrust and change it while phasing. We compared both methods after running phasing on identified SNVs and indels by Clair2 and benchmarked how trust or distrust GT will affect the results compared to the GIAB gold standard (**Supplementary** **Fig S4)**.

We compared the SNVs and indels identified genotype between Clair2, WhatsHap genotype, WhatsHap phasing trust, and WhatsHap phasing distrust (where WhatsHap implicitly re-genotype identified SNVs and indels) across three technologies HiFi, CLR, and ONT (**supplement Fig** **S4**). Furthermore, we found that all the methods achieved F-measure greater than 88% (**Supplementary** **Table** **S3**), and Clair2 achieved the most accurate results across all technologies CLR (99.70%), ONT (99.76%), and HiFi (99.81%). Subsequently, when we used trust GT with WhatsHap phasing, it achieved the second-best results for all technologies **(Supplementary Table** **S5**).

## Structural Variants

We use Sniffles version 1.0.12 to detect SVs 3 reads are the minimum requirement to identify an SV (this parameter can be changed from the configuration file). Additionally, we report all reads used to identified SVs “--num_reads_report -1 --genotype”. For benchmarking we used truth set data from GIAB and compared it using Truvari using default parameters and set “--passonly --giabreport --pctsim=0 --multimatch”.

## Phasing

To benchmark phasing, we used both WhatsHap stat and compare algorithms with GIAB data to get the number of phased SNVs, indels, and phased blocks, likewise, calculate Hamming and switch error rate.

## Methylation

For capture data, from [Gilpatrick, T. *et al.*](http://paperpile.com/b/LV8LI2/hEi9) we compared the methylation events that we detect with what already reported in their paper, by intersecting the coordinate using bedtools intersect.

# Datasets

# HS1011

ONT (17.5x) coverage with 6,766 mean read length and N50 (14,588)

# Figures


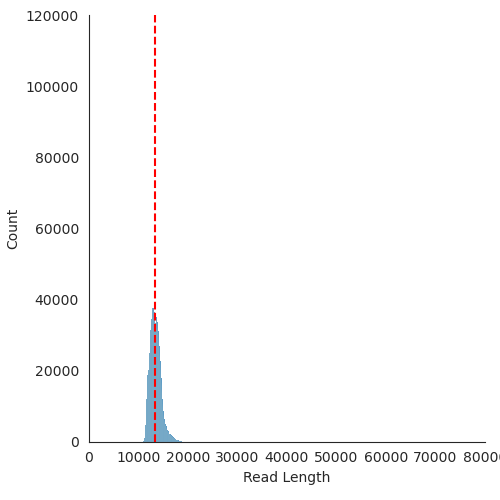


**Fig 1**: HiFi read length distribution.


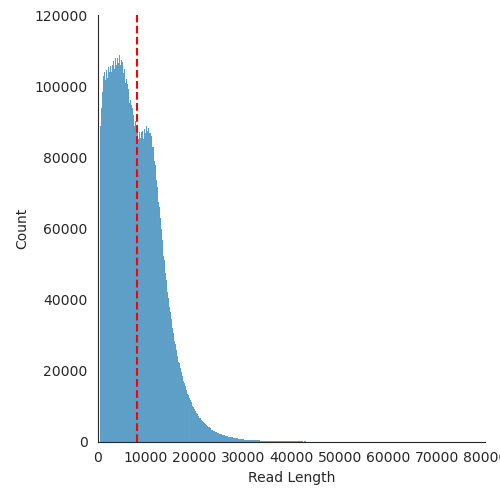


**Fig 2**: CLR read length distribution.


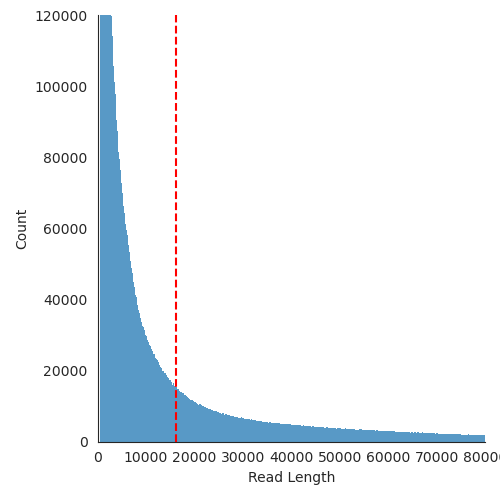


**Fig 3**: ONT read length distribution.


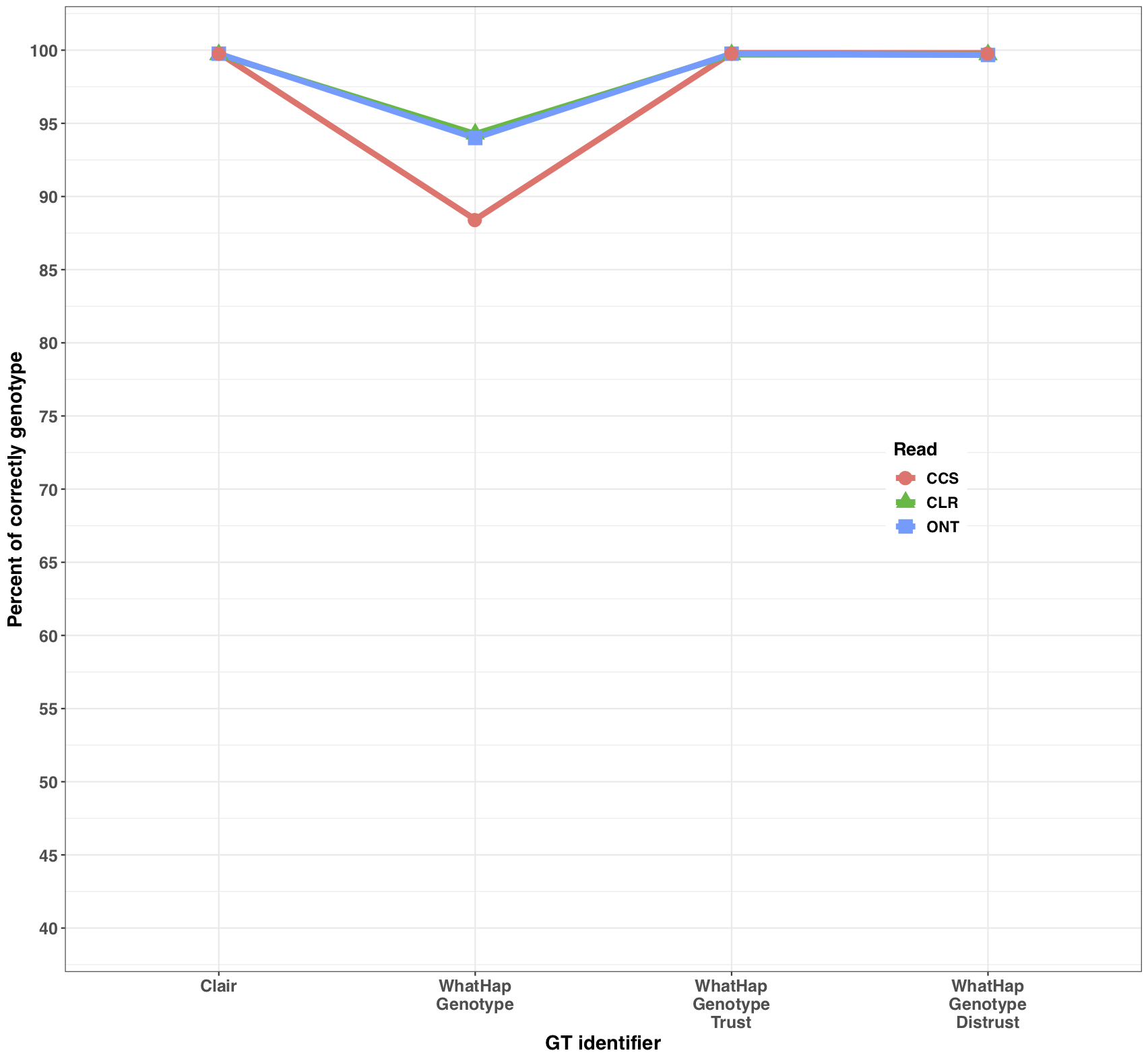


***Fig 4.*** *Genotype identification comparison using different algorithms/tools, across different technologies CCS/HiFi, ONT, and CLR.*


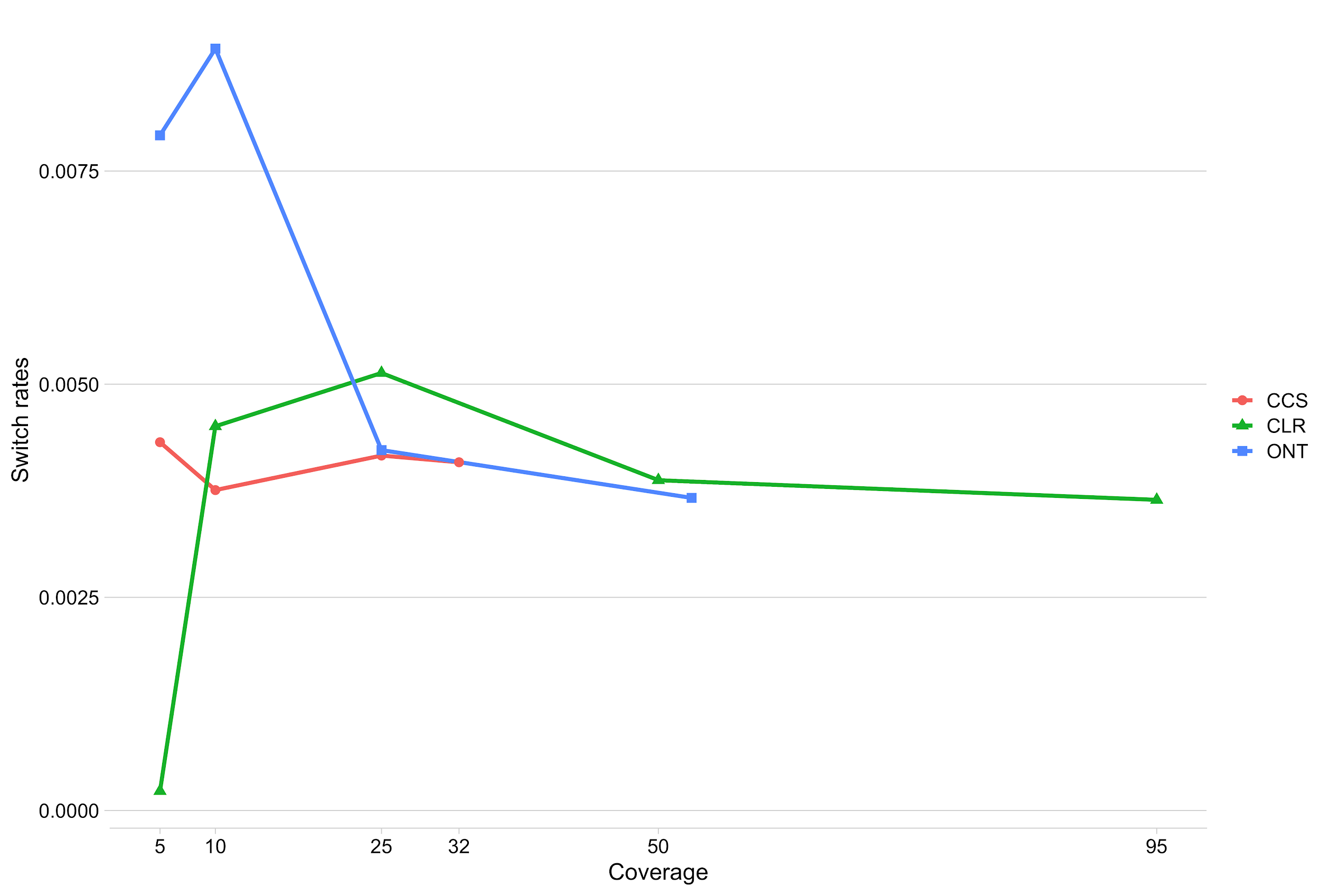


***Fig 5.*** Phasing switch error rates comparison between different technologies CCS/HiFi, ONT, and CLR.


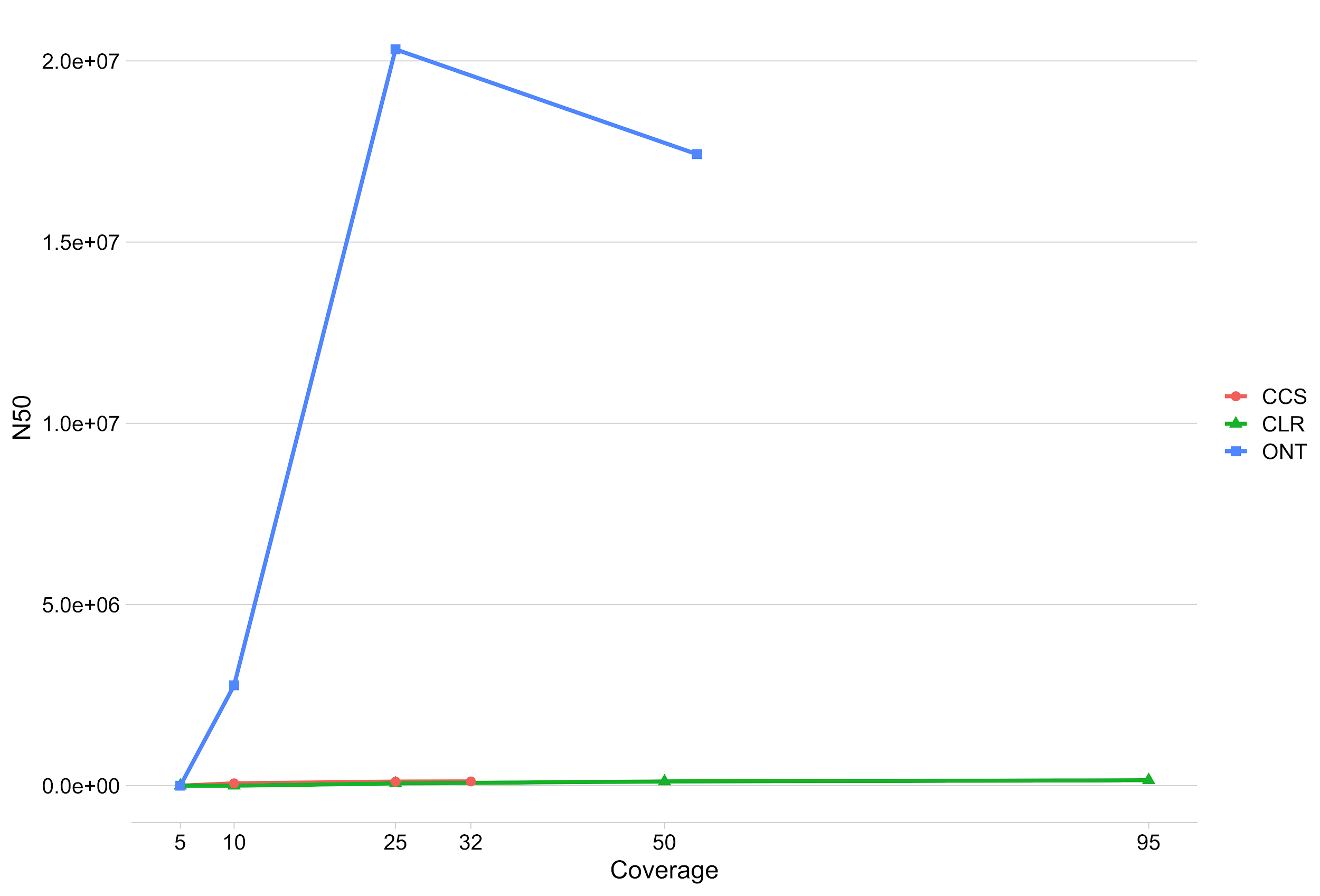


***Fig 6***. Phasing N50 comparison between different technologies HiFi, ONT, and CLR.


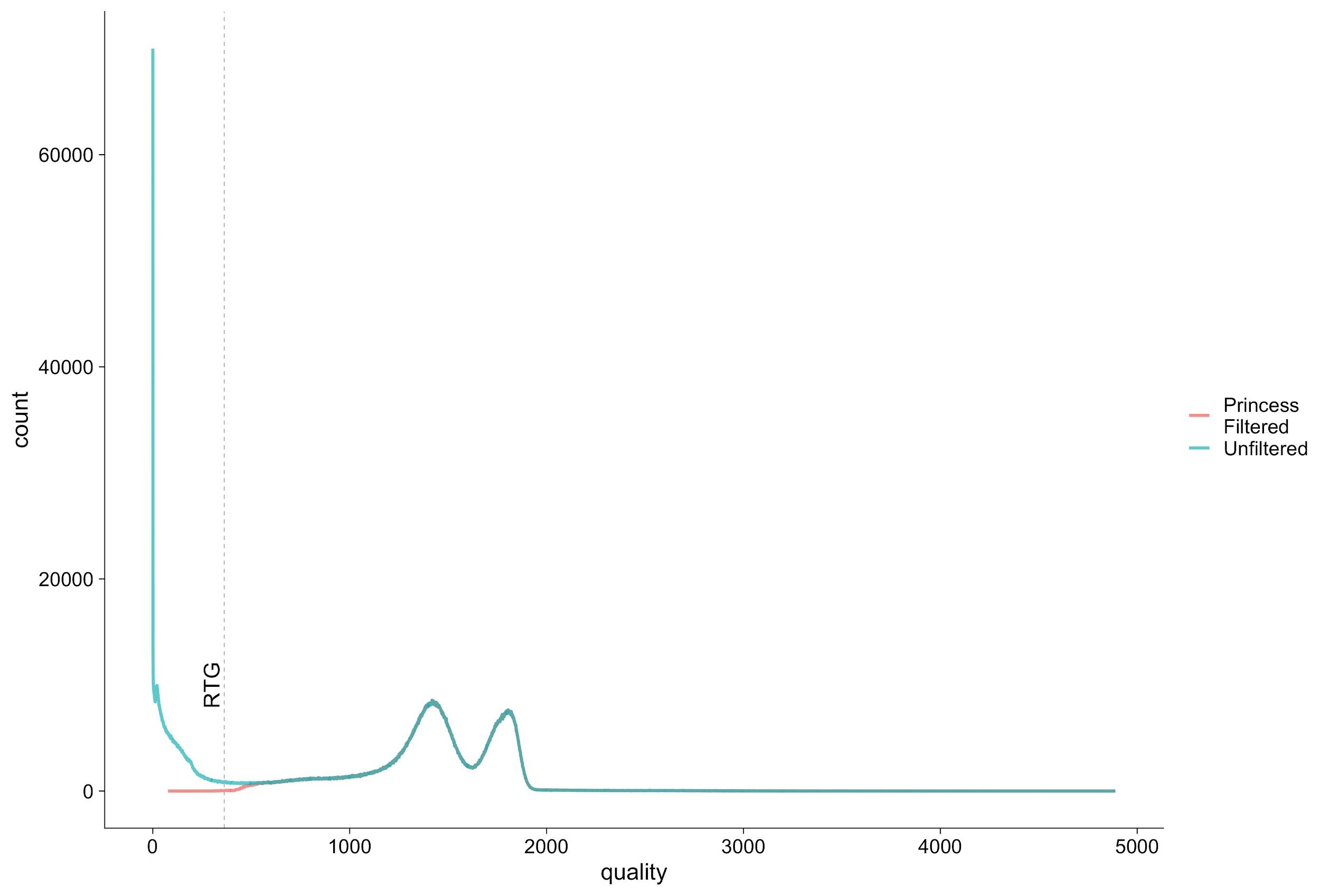


***Fig 7***. HG002 SNVs and indels quality value distribution for insert size 15kb before and after using PRINCESS filter, the red bar shows the RTG suggested filter threshold.


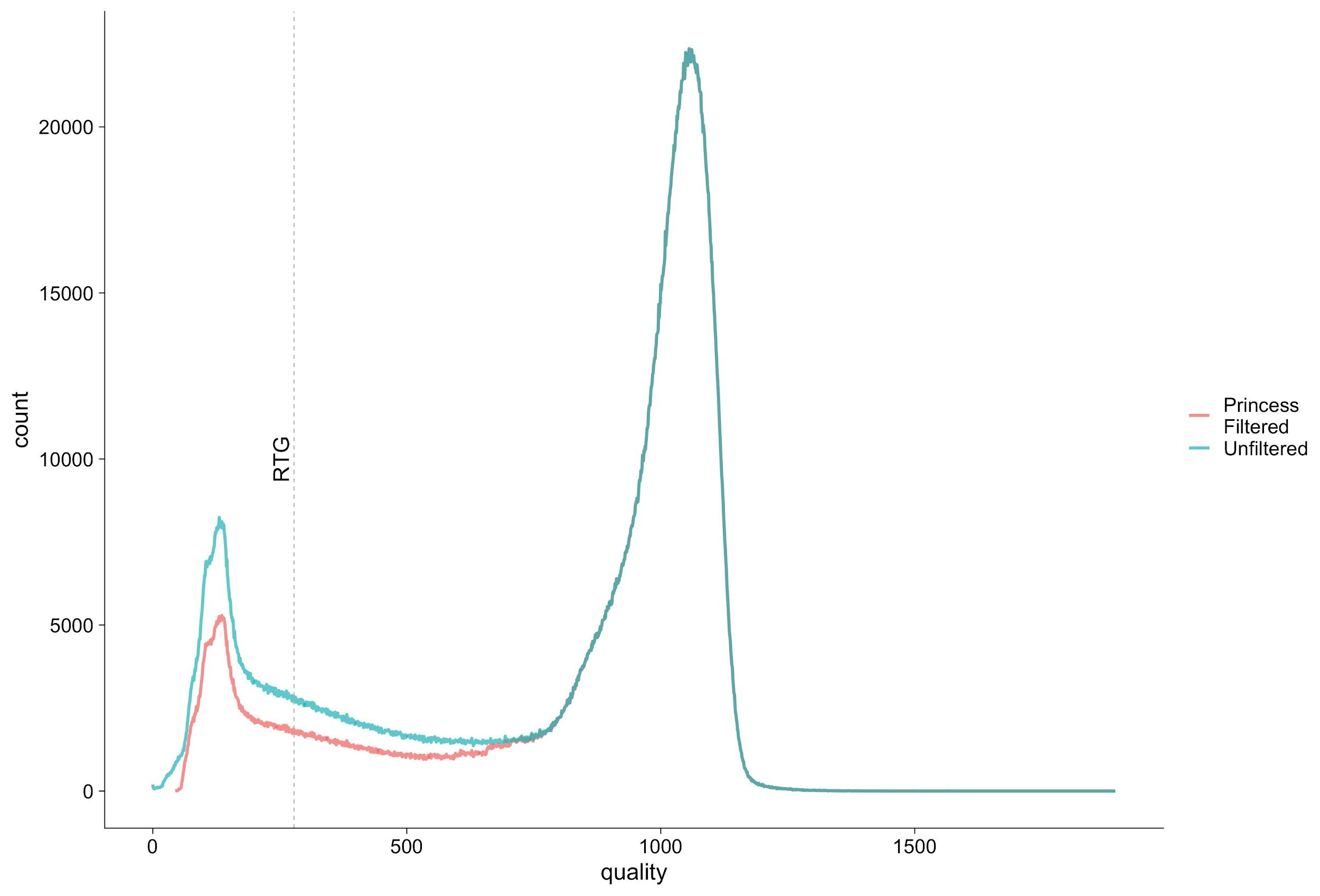


***Fig 8***. SNVs and indels quality value distribution for insert size 19kb before and after using PRINCESS filter, the red bar shows the RTG suggested filter threshold.


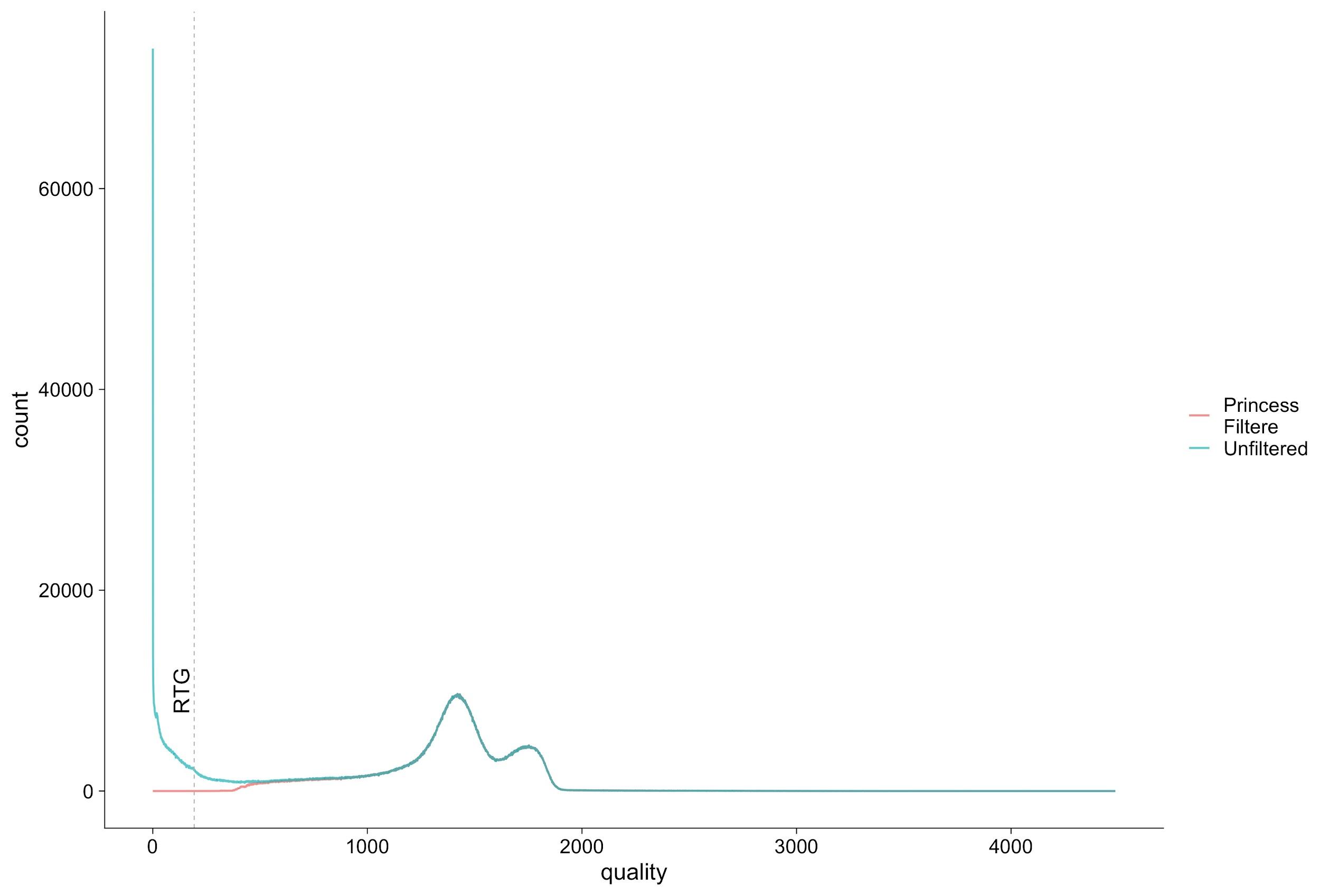


***Fig 9***. SNVs and indels quality value distribution for insert size 20kb before and after using PRINCESS filter, the red bar shows the RTG suggested filter threshold.


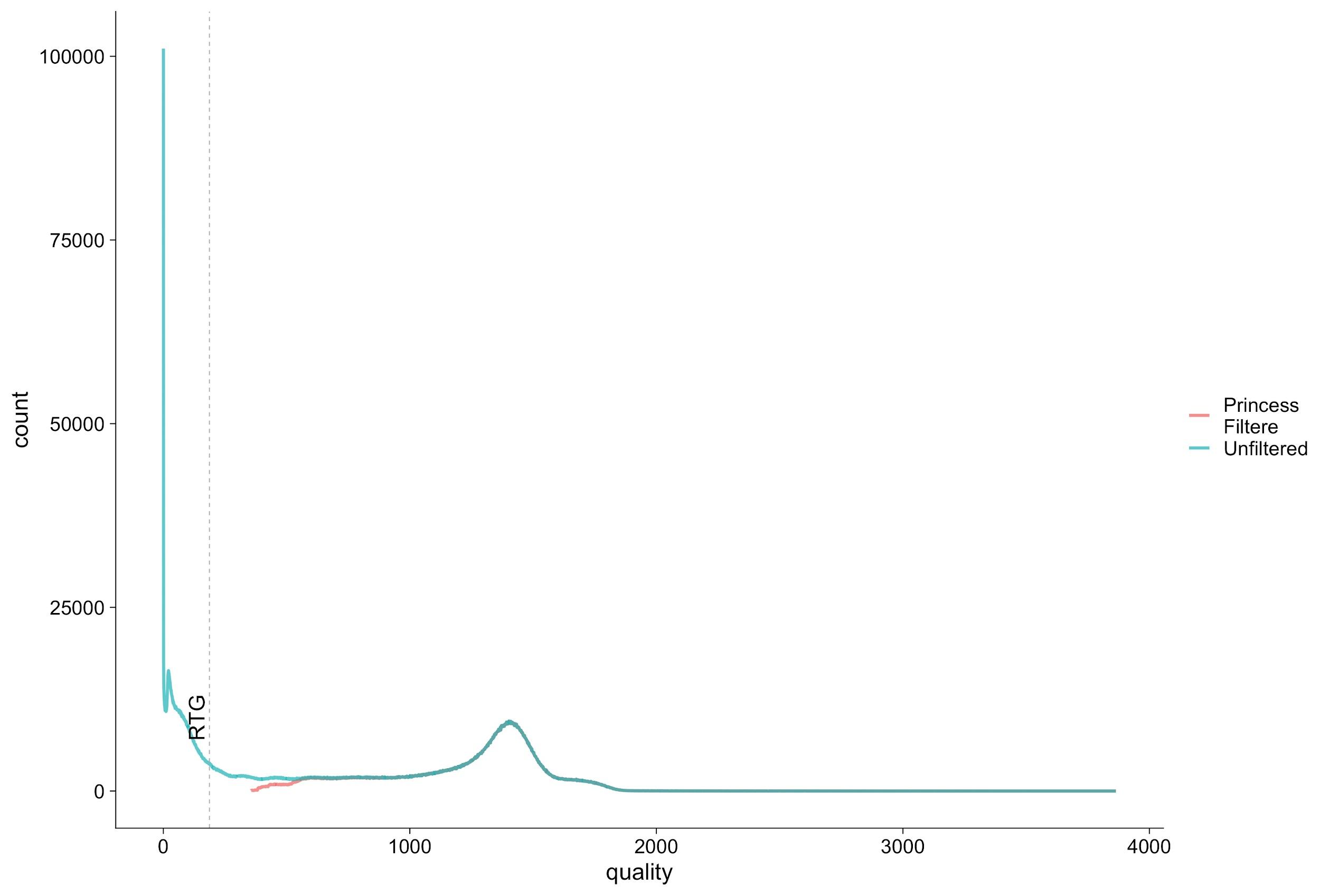


***Fig 10***. SNVs and indels quality value distribution for insert size 25kb before and after using PRINCESS filter, the red bar shows the RTG suggested filter threshold.


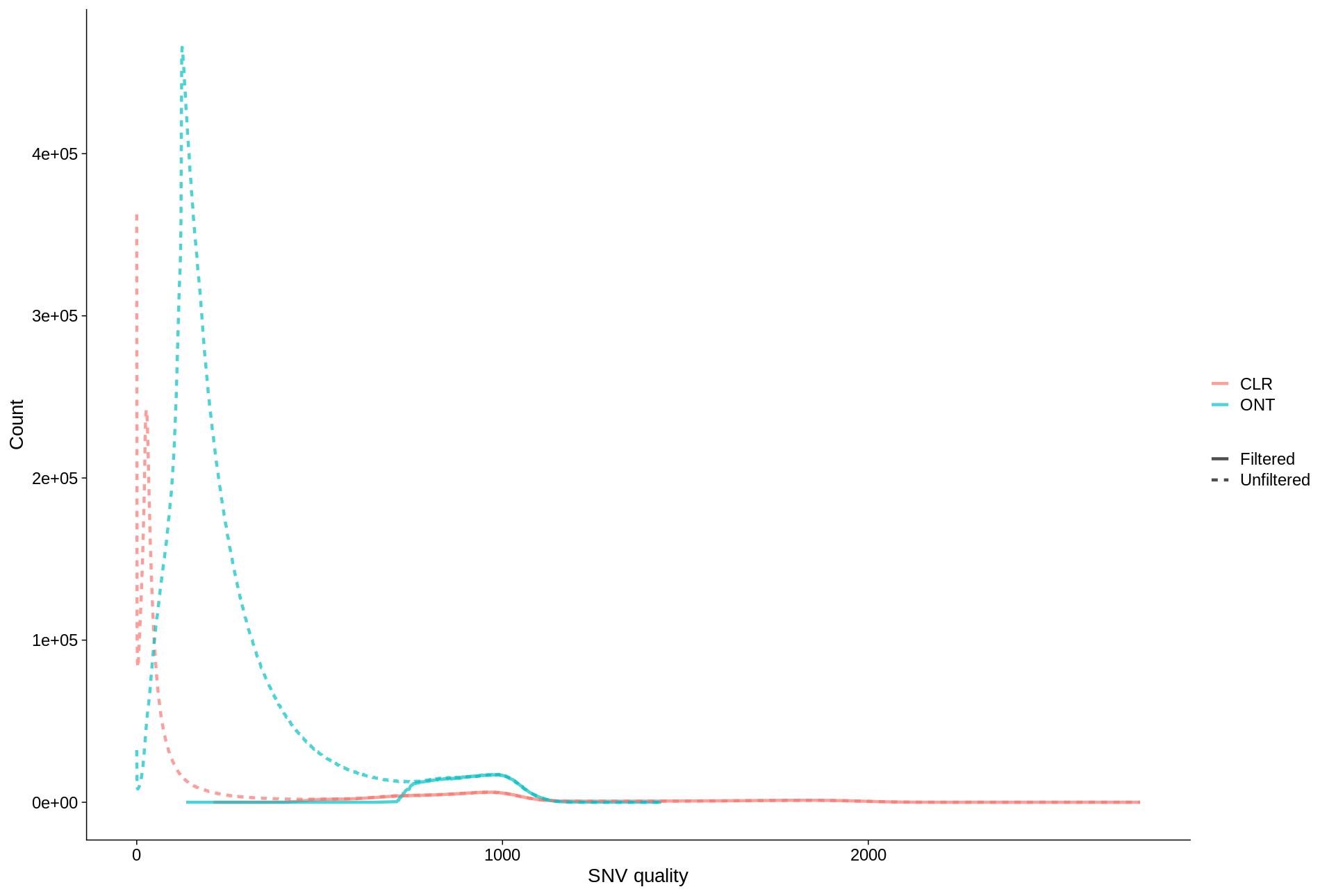


***Fig 11.*** *Comparison between SNVs and indels quality before and after PRINCESS filtering, for both CLR and ONT data for HS1011.*


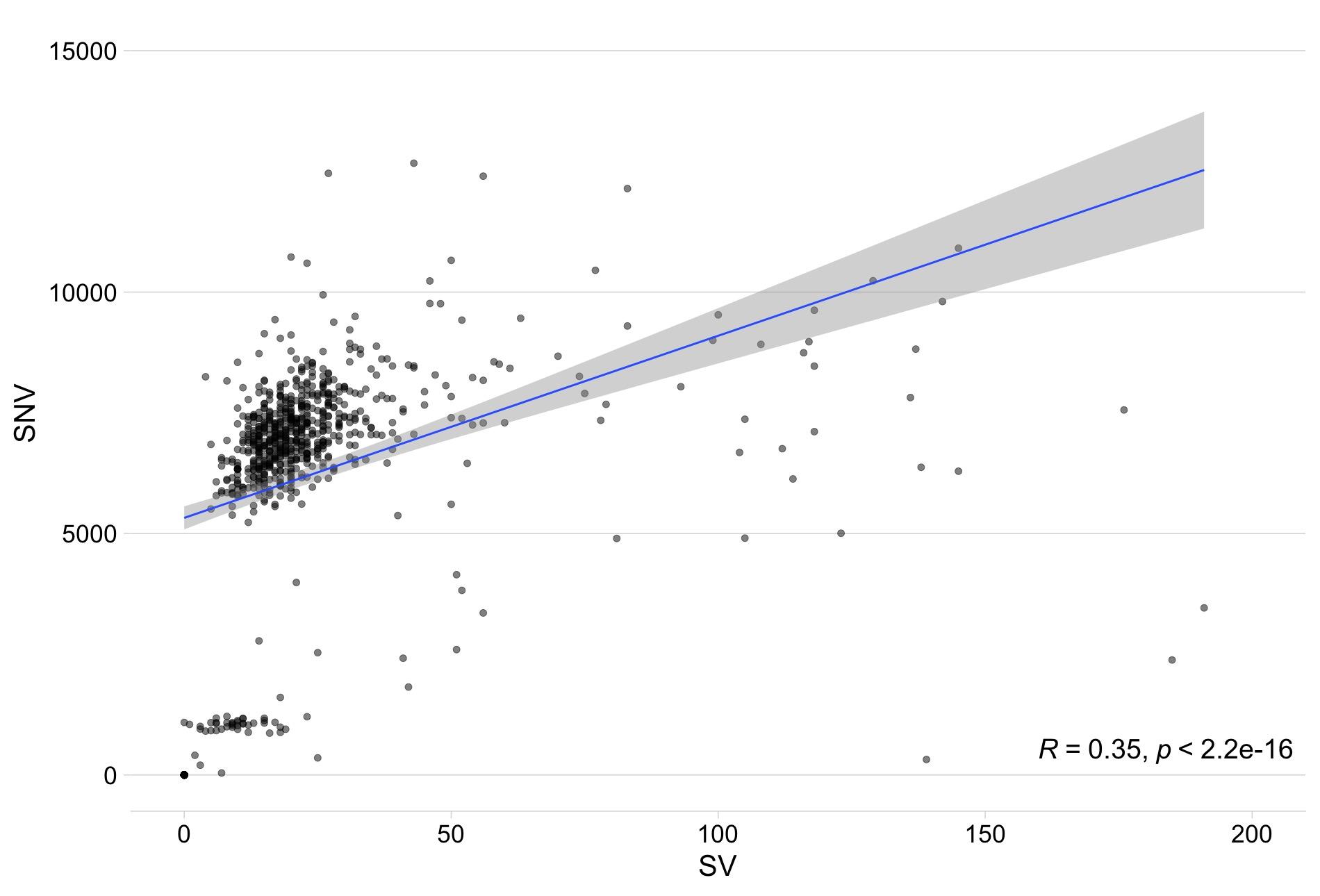


***Fig 12.*** *The relation between the number of identified SVs vs SNVs and indels across 4Mbp windows, using Pearson method to calculate correlation coefficients.*


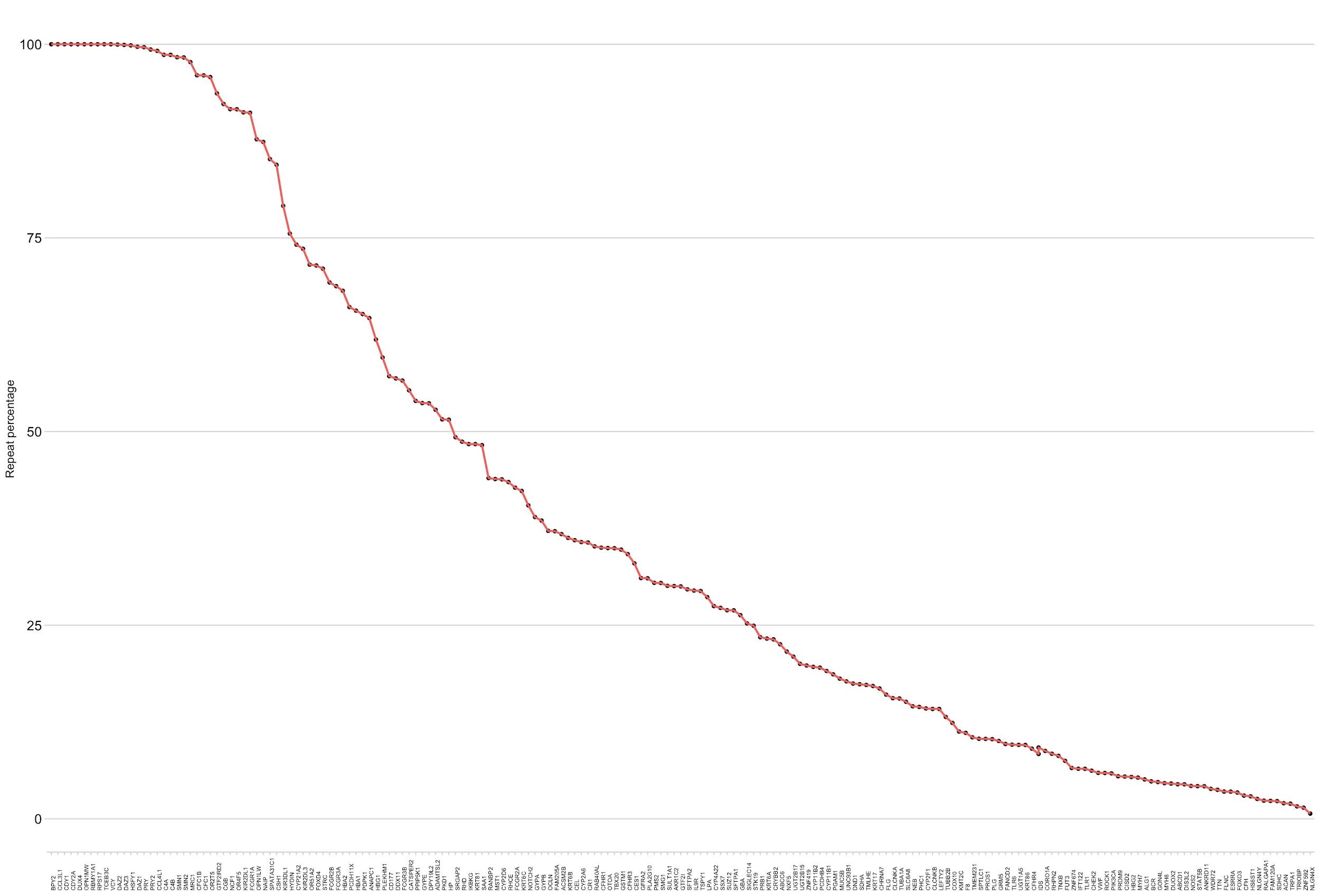


***Fig 13***: Percent of repeats per gene, genes are on x-axis y show the percent of repeats in the corresponding gene.


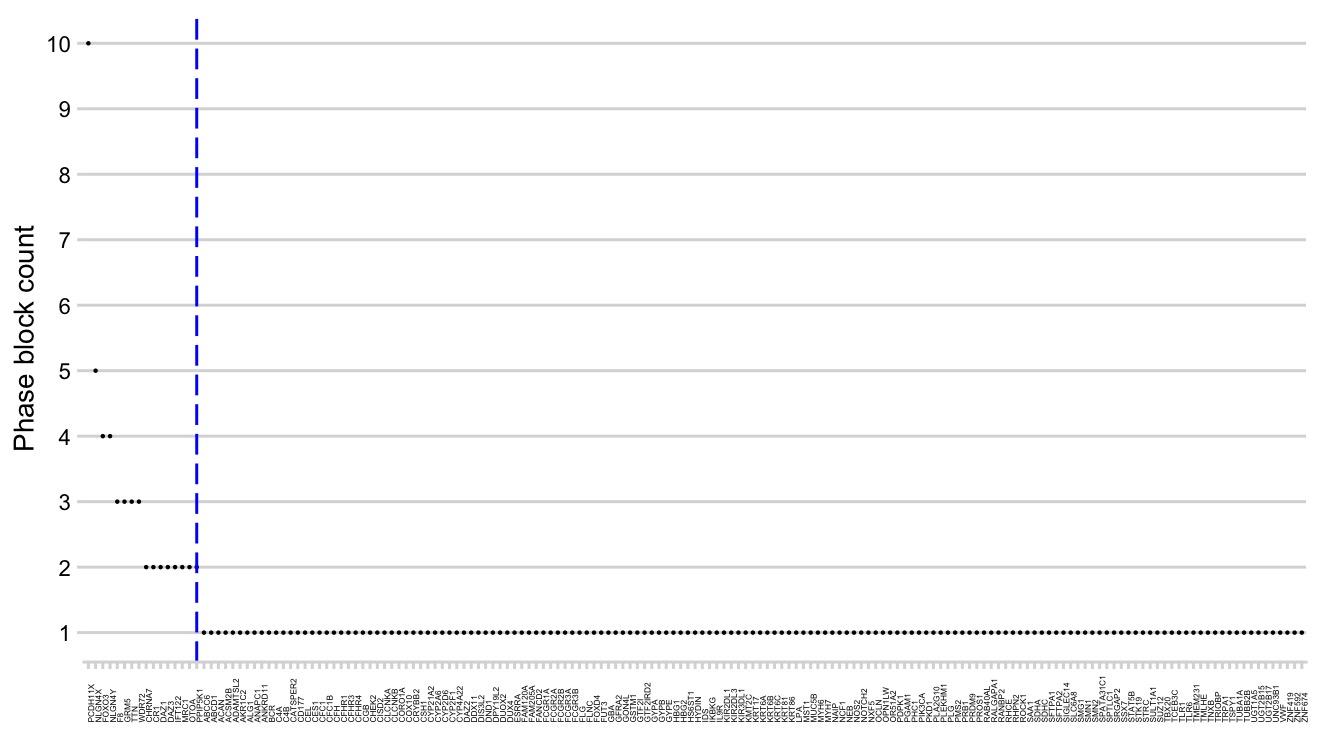


***Fig 14***: Number of phase blocks per genes, X-axis shows the genes named and the Y-axis shows the number of phase blocks, the blue bar shows the difference between genes with more than one phase block and rest of genes.


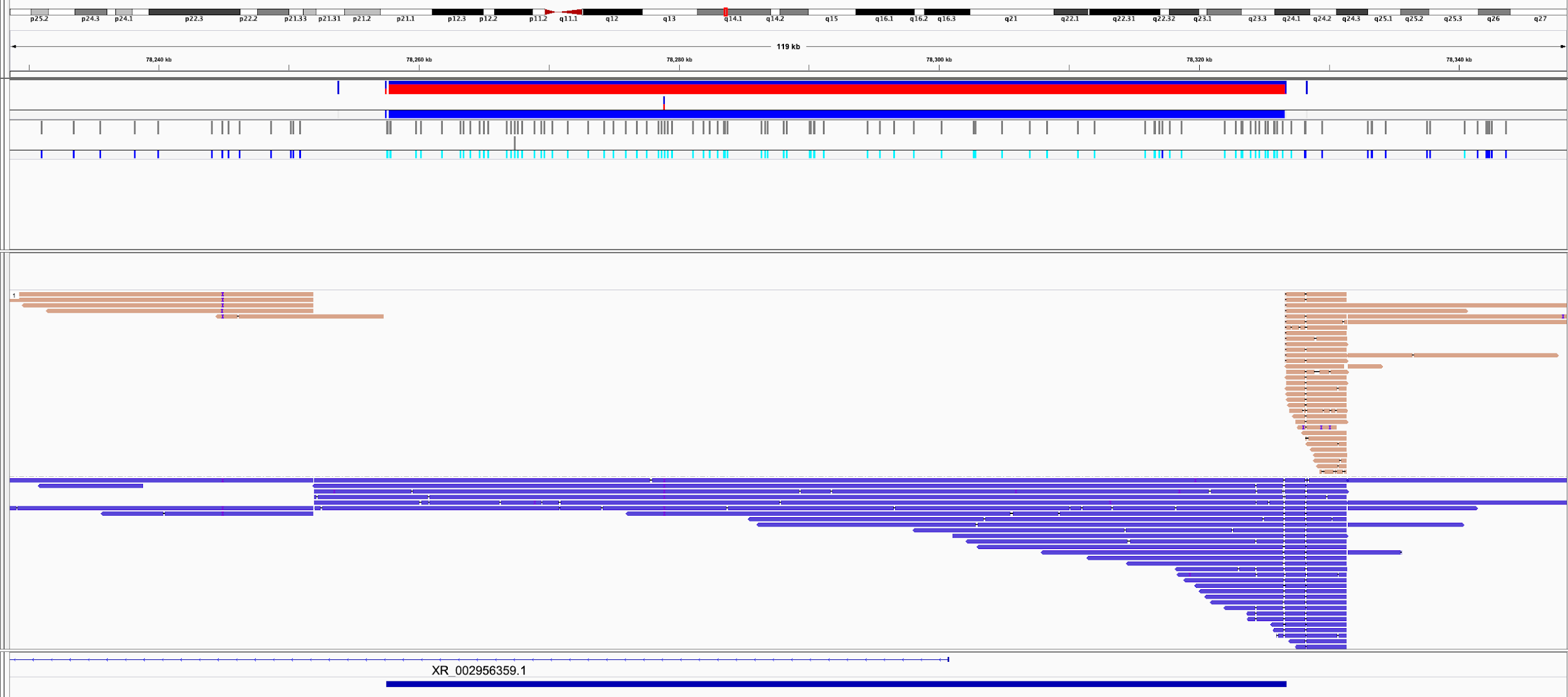


***Fig 15:*** Capture data GM12878 showing a deletion of ~78Kb using Minion.

*
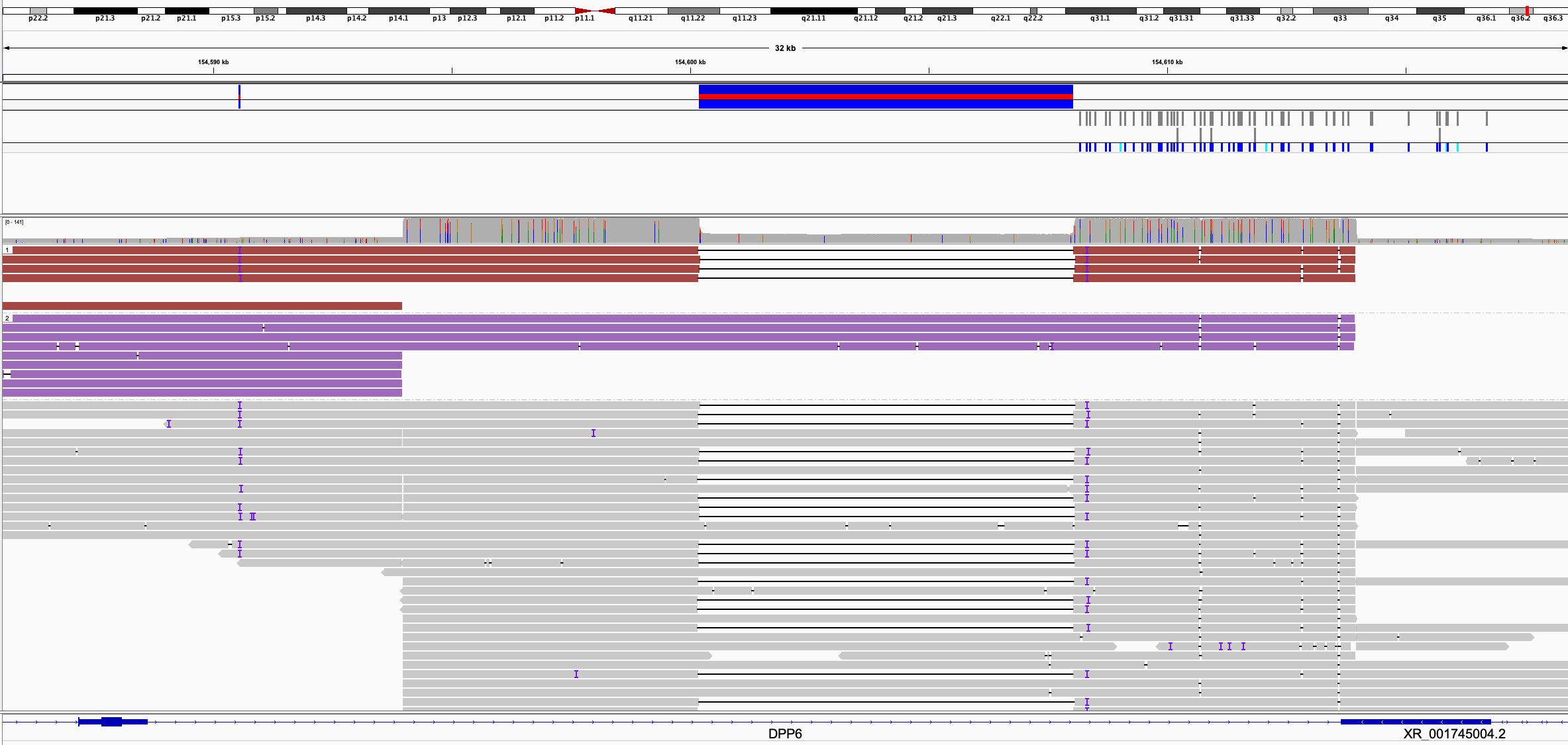
*

***Fig16***: Shows a 32kbp long deletion in sample MdaMb231 based on MinION sequencing data


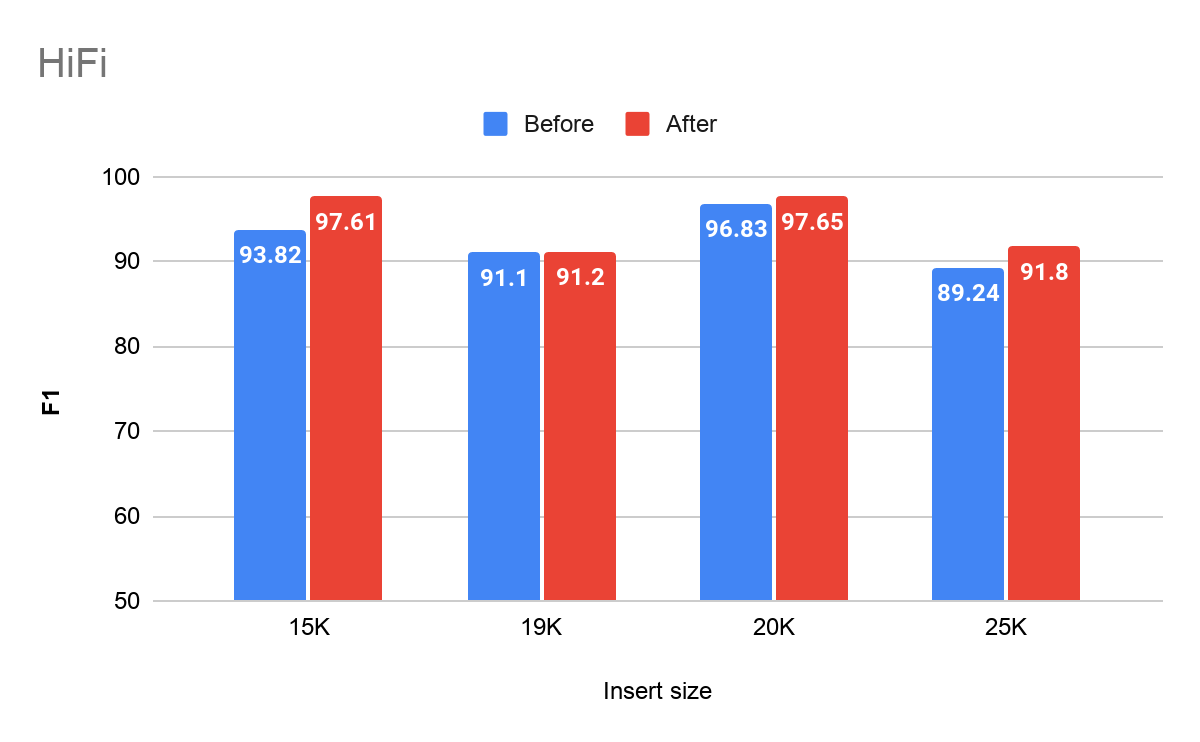


***Fig 17****:* effect of PRINCESS filtering method on F1 score using different HiFi insert size, on the X-axis the insert size, on the Y-axis the F1 score blue before applying filter and red after applying the filter.

***
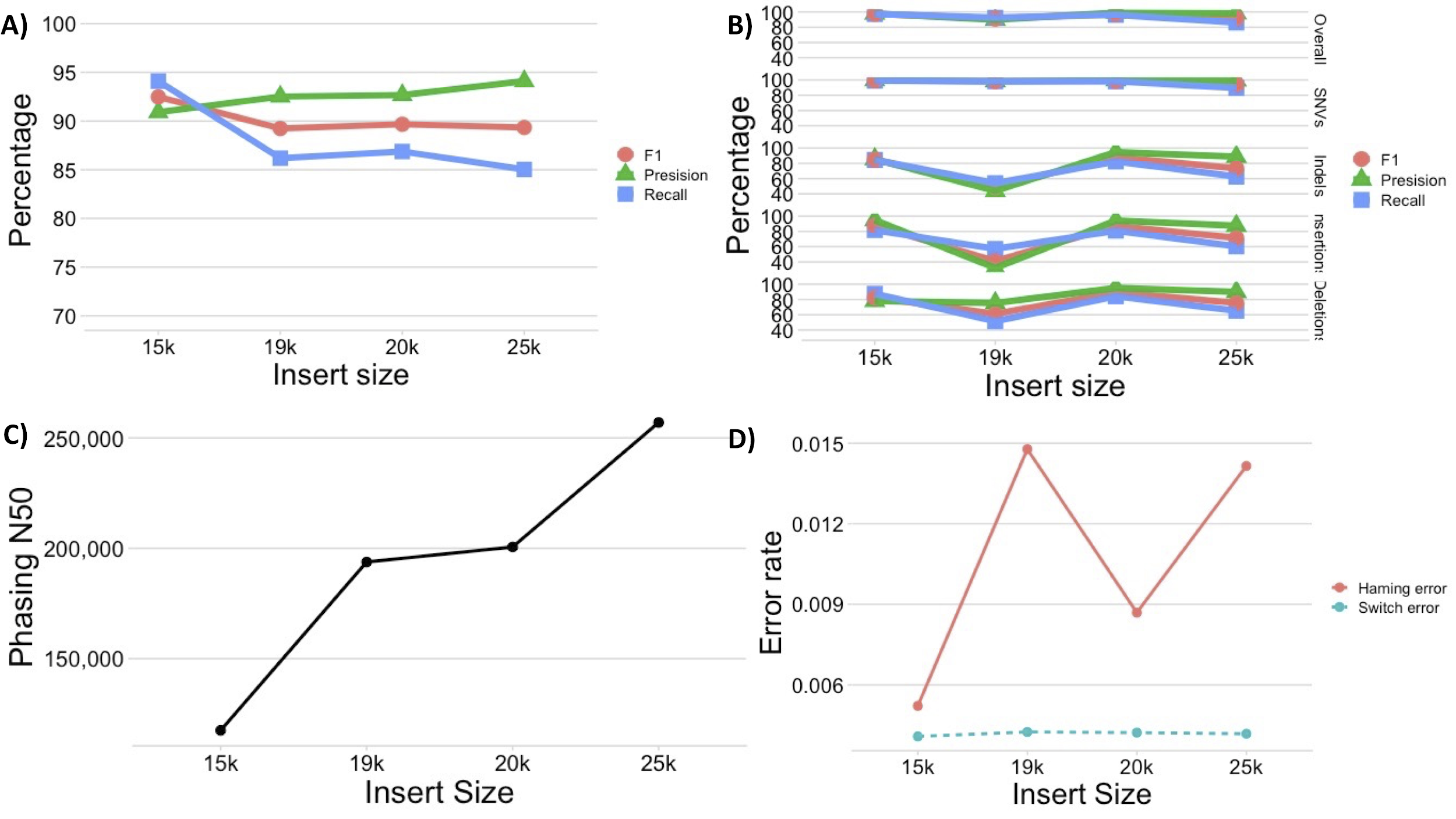
***

***Fig 18:*** HiFi variant detection comparison between different insert sizes **A)** Structural Variant identification between HiFi different insert sizes **B)** Single Nucleotide detection comparison. **C)** Phasing N50 comparison. **D)** Hamming and Switch error rates to insert size.

***
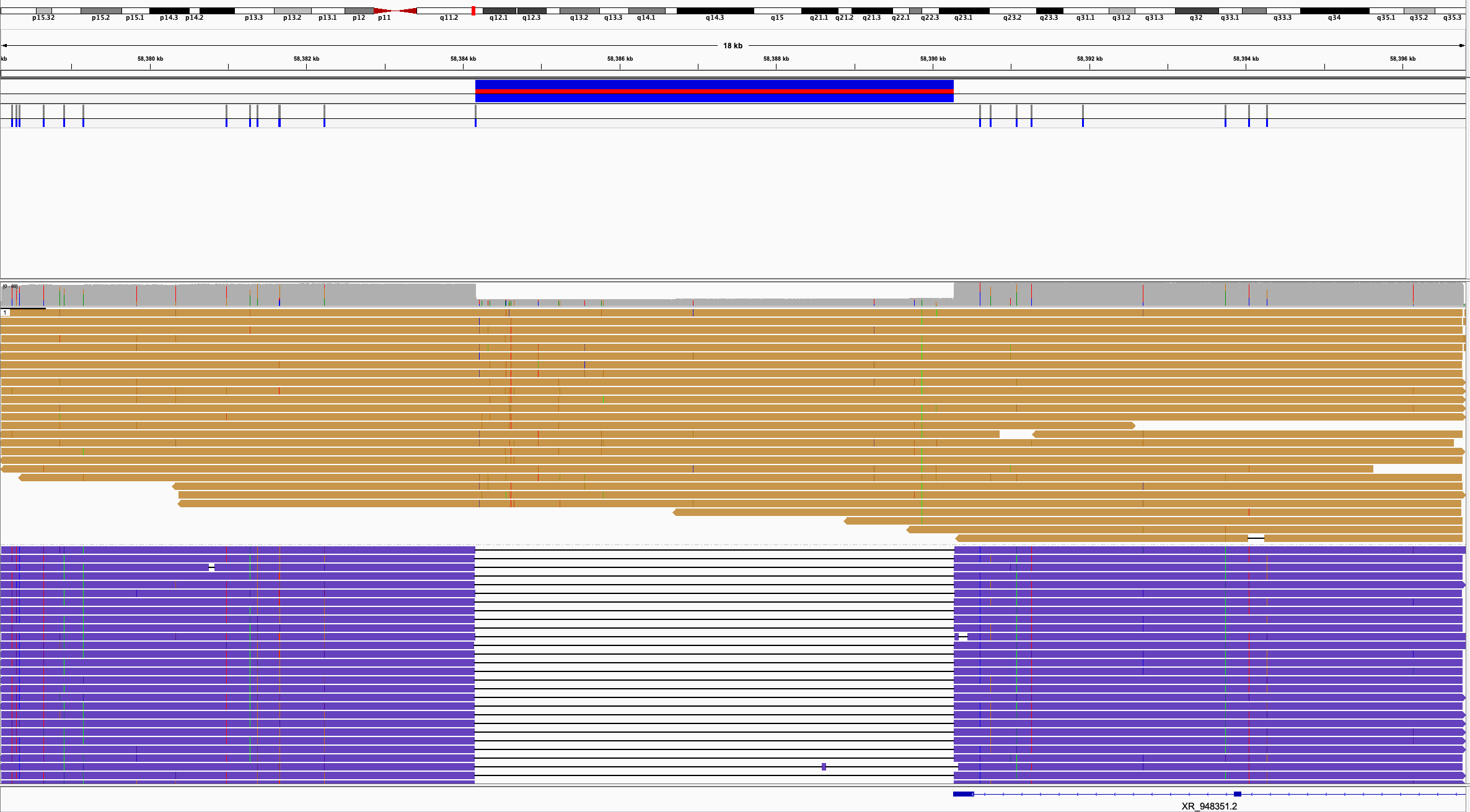
***

***Fig 19****:* A 6,107 bp heterozygous phased deletion on chromosome 5 in the MDA-MB-231 sample.
