# Supplementary material for: PRINCESS: comprehensive detection of haplotype resolved SNVs, SVs, and methylation
Source: Genome Biol. 2021 Sep 14;22:268. doi: 10.1186/s13059-021-02486-w (PMC8442460; doi:10.1186/s13059-021-02486-w)
Supplement: Supplementary file 21 — Additional file 21. Review history. [file 13059_2021_2486_MOESM21_ESM.docx]

Review History

**First round of review**

**Reviewer 1**

**Were you able to assess all statistics in the manuscript, including the appropriateness of statistical tests used?**

Yes, and I have assessed the statistics in my report.

**Comments to author:**

The authors present a framework for variant calling with long reads from SNP to SV. Despite the elite title of princess, this framework wrests variant analysis from long-read bourgeois. By implementing best practices into a pipeline that can be easily used by researchers not familiar with long-read analysis, this tool can have substantial impact on the field.

Developing a robust pipeline for data analysis is not trivial and merits publication. However the primary concern with the manuscript is the description of SNV calling, phasing, and SV calling results as products of the princess method, rather than outcomes of the individual methods. Figure 1 mentions parameter estimation, and it seems this is the critical step for improving over naive execution of the individual steps.

What is done in the parameter estimation steps for SNV? Nothing seems to be mentioned in the supplement other than tuning to different data types.

How do the phasing results differ from what would be output by whatshap with default parameters, or parameters a reasonably experienced researcher would use?

A description of the novel filtering method seems to be missing.

Are the SV calling results any different from a standard run with sniffles?

The methods show slight changes in the alignment based on the input data (e.g. ONT), but no other parameter tuning. Other methods (e.g. pbmm2 for pbsv) have considerable tuning of gap parameters for SV discovery. Is this done?

Minor concerns:

The phasing of SVs is done with haplotagged reads. First, the description of how the phasing is done, "Princess-sub tools use this information to produce a new VCF file with two extra fields. PS field, which indicated the phasing block and a CONFLICT field which give info if there is a conflict between the sequence reads while identifying the PS value." is not sufficient. How is this information used? Are SVs called again with haplotagged reads? Are the called SVs genotyped with the haplotagged reads? If it is the latter, is there a reason for not calling SVs with the haplotagged reads?

What is the take-home message of Figure 4a? Is there a difference from other genomes to be aware of? Does it show success of the method relative to other approaches?

Figure 4b is hard to understand:

There seems to have different point densities. Is this a variable?

Mappability here refers to short read data?

A more intuitive (to me) plot would be percentage of bases with at least 1 read mapped. And more informative than that would be the percentage of bases with enough coverage to make a call in.

The x axis should state "GIAB mapability score" or something more descriptive.

There are a number of awkward wordings or phases that could be simplified. For example, it is good to focus on removing verbage for clarity:

P3, line 67 "to tune the default parameters for an individual species or for the sequencing technology itself" -> "to tune the default parameters for different species or sequencing technologies".

P3 line 68: "Thus it is hard to fully leverage the LRS data set and this is also shown in multiple of the current studies..." -- Perhaps: "Given the complexity for data analysis, many current studies only focus on limited findings from LRS such as SV identification [6] or methylation [32].

p6, L126, "Alongside the detection of the variations" -> "Alongside the detection of variants"...

p7, l140 "We further investigated the impact of different" … -> Just state the result here, "The phasing N50 increases from 140 to 250kb when increasing HiFi library from 15 to 25kb" or something like that.

p7 , 154 "investigated how this SV phasing" -> "investigated how SV phasing"

p8,171 -> changing tense (tense is changed a lot, for example on page 9 we first assess then princess showed, then sensitivity increases", … Present tense is often preferred.

p9 194 "Next, we investigated the concordance of our SV call set with the reported results" -> Just give a label to the MDA-MB-321 callset you are comparing to, and reference that specifically.

p11 "Using princess we could identify 4,156,673 SNVs … as expected the majority of these are substitutions" -> I'm probably wrong, but I've always assumed SNV strictly was used for a substitution of one base for another, not an indel.

p12 l238"further identified 20,979 SV" -> SVs

That is the limit of what I have the time to go through now. I would recommend feedback from a colleague for the remainder of the document.

**Reviewer 2**

**Were you able to assess all statistics in the manuscript, including the appropriateness of statistical tests used?**

Yes, and I have assessed the statistics in my report.

**Comments to author:**

In this manuscript, the authors present PRINCESS, a workflow for processing long-range sequencing data, which includes SNV, SV and methylation calling, with phasing. The authors correctly identify an important gap in the literature, as to this reviewers knowledge, such a single-stop approach does not exist.

The manuscript is let down by dozens, if not >100 grammatical and formatting errors, which raises concerns regarding the accuracy of the software and the general level of care taken throughout the whole approach. A major revision addressing these changes will help a subsequent review to focus on the science.

From the science that could be assessed, the authors did not convincingly demonstrate whether the performance with regard phasing accuracy, or genotyping accuracy is indeed state of the art. The methylation results are anecdotal and that these data are phased in conjunction with SNV and SV is not presented. The supplementary data were absent from the submission, so was unable to be assessed. Despite this, there were several sentences linking to supplementary results that were under-explained/under-summarised in the main text.

Major items

1. Given the enthusiasm amongst the LRS community for these technologies to identify all classes of variation, accurately, phased, genotyped and with methylation data, I was surprised to see the rates of mutation detection (SNV and SV), rates of phasing, and rates of phase errors lower or higher than expected - certainly lower than the abstracts conclusions would suggest. This manuscript would be much improved if these values were benchmarked to previous best practice approaches, to demonstrate that the performance of PRINCESS across all of its categories is indeed class-leading.

2. The methylation results are hinted at on line 199 and then concluded to 'reveal the entire biological picture' on line 204. As presented, these claims are entirely unjustified. I recommend you summarise these findings in the main text, highlight specific examples where this information is useful (eg hypermethylation of a tumour suppressor gene previously reported to be important in these cell lines), and demonstration that these data are indeed phased, and that phasing has improved the interpretation of these data.

3. The PRINCESS GitHub homepage barely mentions methylation, so can the software actually process and phase methylation data?

4. Phasing of SV and SNV: The conclusions on line 163-4 are unjustified. Phasing rates of 38-90% do not satisfy this reviewer as being high accuracy. These claims may be strengthened by comparing to state of the art methods, and appropriately reserved language. Adding specific examples where phasing helps resolve the pathogenicity/otherwise of alleles found to be in cis, or in trans.

5. Accuracy of variant calling within the 193 difficult genes. Tackling these difficult to sequence genes is an important contribution of the paper. However, the quality and accuracy of the genotypes within these genes are not reported. The proportion of heterozygous sites within these difficult to sequence regions appears far higher than expected (ie very few homozygous alt sites) than typical genes. Line 226 suggests these 'does not seem to be affected' which is not supported by the data. In short - please provide evidence that these variants are real, preferably with orthogonal data (eg 2 or more LRS methods), and at a minimum, focussing on the quality attributes (AD, base quality, ...).

6. HS1011 analysis: I found this difficult to follow, and I recommend that you focus on solving the diagnostic case in a succinct section, with a discussion the cis/trans alleles and how this information has helped diagnose the case. Are there any links to aberrantly methylated genes in CMT that your LRS data could shed light on in this case? The transition from candidate gene, SH3TC2, to the other 3 genes in Figure 5 could be improved; suggest you merge fig 4A and fig 5A together to speak to the diagnosis, and then Fig 4B,4C,5B-D in the same figure to speak to the exploration of difficult regions.

7. Translocations: 3.49% of SVs in HS1011 were translocations; I would expect this to be close to 0 in a germline case, unless that translocation affected the candidate gene, suggesting these are false positive calls, or perhaps low-level mosaic calls. Please investigate these.

8. I found the discussion of rates of SV types relative to what's already known from short-read and LRS technologies to be difficult to follow. Eg on L241 and L244-245 speak to two separate contradictions in these data relative to 2 previous studies.

9. If showcasing the capabilities of PRINCESS, Figure 3 is underwhelming, as any short and long-read technology should easily resolve a 6Kb deletion, especially with two variants in trans overlapping the variant. What gene is affected here, and, based on the position relative to this transcript's TSS, you've missed an opportunity to investigate whether any regulatory elements are affected by this deletion, and whether they affect [publicly available] expression data for this transcript. I recommend you reconsider the elements in this figure to more holistically summarise the cancer data analysis.

10. L432 - under what ethics was this patient consented? You indicate that this patient's data was shared to SRA. dbGaP or EGA seem a more secure choice for human genome data, so is SRA consistent with your ethics? What is the accession number?

11. The coverage subsampling analysis was hard to follow, with most of the justification for doing this appearing much later in the discussion. I recommend rewriting this section in a more proactive and justified way.

Minor items

*	This manuscript has dozens of grammatical and formatting errors throughout.

*	Inconsistent spacing and commas before and within parentheses. Eg L116 twice, and page 6 in several spots

*	Supplementary vs Supplement throughout, followed by consistent capitalisation of Table or Figure or Section and spacing, eg L116, L124, L120, dozens more)

*	Inconsistent spacing before citations, eg L63-65

*	Figure 1C legend doesn't explain the role of coverage nor that subsampling analyses were performed

*	Throughout, SNV and SV are mentioned, but not indels. If you have bundled indel performance into SNV performance than I recommend you make this clear at the start. Further, indel errors appear common in ONT data (and perhaps other LRS), so combining SNV with indels may explain why the reported SNV accuracy relative to NIST is lower than i would expect for only SNVs

*	L144 replace 'succeeded by'

*	Inconsistent use of commas and confusing use of two values, eg L147 - L151 or L159 are better examples of reporting multiple numbers

*	L139 and L140, summarise what you found

*	L143 and L154 both start with next; suggest a different approach.

*	L163 'high rate of accuracy'; L206 'high concordance' - as mentioned above, this needs justification

*	L174 is out of place; move to methods.

*	L183 Flongual?

*	L195 - how large were these SVs?

*	Tumorigenetic should be tumorigenic

*	Performances should be performance

*	L109 people should be researchers

*	Variations should be variants

*	Gene symbols should be italicised

*	L269 … stars indicate…

*	L283 was hard to follow

*	L281-282 should be split and explained more clearly

*	L287 how did you get 287 genes?

*	L299 clarify that these are hard to map from conventional short-read seq, or hard to map from LRS.

*	L308, L312 introduce the name of GBA gene with two different names?

*	L328-L629 is unjustified

*	L334 - phasing and methylation is unsupported as noted above

*	L350 typo

*	L352 suggest to end with 'de novo variants'

*	L3863-384 is repetitive

*	L385 should be rephrased

**Authors Response**

**Point-by-point responses to the reviewers’ comments:**

We would like to thank both reviewers for their helpful comments and remarks. We have responded to every question from them below and have revised the main text to improve the language and the clarity. We highlighted the changes and our responses in blue.

Reviewer #1:

The authors present a framework for variant calling with long reads from SNP to SV. Despite the elite title of princess, this framework wrests variant analysis from long-read bourgeois. By implementing best practices into a pipeline that can be easily used by researchers not familiar with long-read analysis, this tool can have substantial impact on the field.

Developing a robust pipeline for data analysis is not trivial and merits publication. However the primary concern with the manuscript is the description of SNV calling, phasing, and SV calling results as products of the princess method, rather than outcomes of the individual methods. Figure 1 mentions parameter estimation, and it seems this is the critical step for improving over naive execution of the individual steps.

*We thank the reviewer for her/his suggestions.*

1.	What is done in the parameter estimation steps for SNV? Nothing seems to be mentioned in the supplement other than tuning to different data types.

*PRINCESS sets predefined parameters according to the sequencing technology (PaBio CLR , PacBio HiFi, ONT). Additionally, we developed a filtering algorithm that filters based on the distribution of the quality values from Clair2. This method was derived from HG002 and slightly adjusted for each of the technologies. We show this benchmark/test on Supplementary table 1 and Figure 2A. Using this filtering algorithm (Supplement 2.1.1) we enhanced the F1 score for e.g. HiFi data as an example from 93.82% to 97.61%.*

2.	How do the phasing results differ from what would be output by whatshap with default parameters, or parameters a reasonably experienced researcher would use?

*As we mentioned in the previous question, filtering out low-quality variants will increase precision and recall. As a result, it will enhance phasing (which is unique to PRINCESS). Additionally, we compared if different genotyping algorithms will affect the accuracy of the called variant. This is important since e.g. Whatshap only considered heterozygous variants to be phased. We compared how the genotype impacts the phasing itself by using the genotypes from SNV calls from Clair compared to the WhatsHap genotype algorithm. We relied on the parental phasing as a truth set. The genotypes from the filtered Clair SNV resulted in the highest phasing percentage (PRINCESS genotype accuracy: ONT 99.76% and HiFi 99.80%) compared to the genotyping of WhatsHap itself (genotype accuracy: ONT:93.99% and HiFi: 88.42%). The details are shown in Supplementary Table S2.*

3.	A description of the novel filtering method seems to be missing.

*We thank the reviewer. Quality values of identified SNVs by Clair are distributed in two bell curved shape, to get the optimum result the threshold point between these two curves should be identified (based on https://github.com/HKU-BAL/Clair#pacbio-ccs-data). PRINCESS implements a filtering process that automatically identify the SNVs quality threshold, we compared the quality results for SNVs before and after using PRINCESS filter as well as the RTG benchmark for different technologies (HiFi, CLR, and ONT), we mentioned it in section 2.1.1 supplement material, and we edited it for clarity.*

4.	Are the SV calling results any different from a standard run with sniffles?

*We are running Sniffles with non default parameters, which enables also the calling of SV at very low coverage data sets. We noted previously that Sniffles achieves good results for 12x or higher coverage data sets. Using PRINCESS, this requirement can be lowered to 10x with a F-score of 86%.*

5.	The methods show slight changes in the alignment based on the input data (e.g. ONT), but no other parameter tuning. Other methods (e.g. pbmm2 for pbsv) have considerable tuning of gap parameters for SV discovery. Is this done?

*No, we have not tuned alignment parameters for the mapping of long-reads. PRINCESS follows the default suggestions for minimap2 and sets the recommended parameters based on the input (ONT vs. PacBio). The scientist can also switch to NGMLR alignments, which in general take a longer run time, but are sometimes more precise in certain regions of the genome (e.g. GBA). Likewise, we add parameters that are required to run succeeding tools, like RG for WhatsHap.*

6.	It would be extremely helpful if the authors linked a tiny test case to the github page. This will help debug installations and make sure a test run can go through without having to set one up ourselves.

*We like to thank the reviewer for this suggestion. We added a test HiFi Fastq file and the command in detail to run a complete test case for that file to the GitHub repository.*

Minor concerns:

7.	The phasing of SVs is done with haplotagged reads.

First, the description of how the phasing is done,

"Princess-sub tools use this information to produce a new VCF file with two extra fields. PS field, which indicated the phasing block and a CONFLICT field which give info if there is a conflict between the sequence reads while identifying the PS value." is not sufficient.

How is this information used?

Are SVs called again with haplotagged reads?

Are the called SVs genotyped with the haplotagged reads?

If it is the latter, is there a reason for not calling SVs with the haplotagged reads?

*We are using the phased SNV to tag the mapped reads (using Whatshap). Sniffles is run to report the read names that support each of the SV. Subsequently, PRINCESS looks up the reads that support the SV and their phasing tag in the bam file to decide if there is a conflict free phasing possible to assign the SV to the individual haplotype of the SNV. Supplementary section 1.6.2 provides more details on this process.*

*This way SNV + phasing can run in parallel to SV calling and thus speeds up the process by a few hours compared to SV calling based on the phased reads. Another disadvantage for using the phased reads is that SV calling would be dependent on the correctness of phasing and sufficient coverage per haplotype.*

8.	What is the take-home message of Figure 4a? Is there a difference from other genomes to be aware of? Does it show success of the method relative to other approaches?

*We removed the figure and combined it with figure 5A to give an explanation about the density of variants in the genome. Figure 4a gives a general overview of variants across this sample.*

9.	Figure 4b is hard to understand:

There seems to have different point densities. Is this a variable?

Mappability here refers to short read data?

A more intuitive (to me) plot would be percentage of bases with at least 1 read mapped. And more informative than that would be the percentage of bases with enough coverage to make a call in.

The x axis should state "GIAB mapability score" or something more descriptive.

*The mappability here indeed refers to the GIAB mappability score and we adopted this axis label to make this clearer. We also thank the reviewer for this suggestion and changed the Figure accordingly. We did not assess the coverage to make a call since this is depending on multiple factors (e.g. homozygous vs. heterozygous variants).*

10. There are a number of awkward wordings or phases that could be simplified. For example, it is good to focus on removing verbage for clarity:

P3, line 67 "to tune the default parameters for an individual species or for the sequencing technology itself" -> "to tune the default parameters for different species or sequencing technologies".

*Changes were made accordingly*

P3 line 68: "Thus it is hard to fully leverage the LRS data set and this is also shown in multiple of the current studies..." -- Perhaps: "Given the complexity for data analysis, many current studies only focus on limited findings from LRS such as SV identification [6] or methylation [32].

*Changes were made accordingly*

p6, L126, "Alongside the detection of the variations" -> "Alongside the detection of variants"...

*Changes were made accordingly*

p7, l140 "We further investigated the impact of different" … -> Just state the result here, "The phasing N50 increases from 140 to 250kb when increasing HiFi library from 15 to 25kb" or something like that.

*Changes were made accordingly*

p7 , 154 "investigated how this SV phasing" -> "investigated how SV phasing"

*Changes were made accordingly*

p8,171 -> changing tense (tense is changed a lot, for example on page 9 we first assess then princess showed, then sensitivity increases", … Present tense is often preferred.

*Changes were made accordingly*

p9 194 "Next, we investigated the concordance of our SV call set with the reported results" -> Just give a label to the MDA-MB-321 callset you are comparing to, and reference that specifically.

*Changes were made accordingly.*

p11 "Using princess we could identify 4,156,673 SNVs … as expected the majority of these are substitutions" -> I'm probably wrong, but I've always assumed SNV strictly was used for a substitution of one base for another, not an indel.

*We changed it to be” 4,156,673 variants (SNVs and indels)” to make this clear.*

p12 l238"further identified 20,979 SV" -> SVs

*Changes were made accordingly*

That is the limit of what I have the time to go through now. I would recommend feedback from a colleague for the remainder of the document.

Reviewer #2:

In this manuscript, the authors present PRINCESS, a workflow for processing long-range sequencing data, which includes SNV, SV and methylation calling, with phasing. The authors correctly identify an important gap in the literature, as to this reviewers knowledge, such a single-stop approach does not exist.

*We thank the reviewer for recognizing the importance of PRINCESS and his /her helpful comments.*

1.	The manuscript is let down by dozens, if not >100 grammatical and formatting errors, which raises concerns regarding the accuracy of the software and the general level of care taken throughout the whole approach. A major revision addressing these changes will help a subsequent review to focus on the science.

*We thank the reviewer for her/his comments and we improved the language of the manuscript.*

2.	From the science that could be assessed, the authors did not convincingly demonstrate whether the performance with regard phasing accuracy, or genotyping accuracy is indeed state of the art. The methylation results are anecdotal and that these data are phased in conjunction with SNV and SV is not presented. The supplementary data were absent from the submission, so was unable to be assessed. Despite this, there were several sentences linking to supplementary results that were under-explained/under-summarised in the main text.

*We thank the reviewer for the question. We are sorry that there was a problem with the Supplement. We checked and couldn't find a problem from our end. We will ensure that the Supplement is submitted with the response.*

*Regarding phasing and SNVs calling, please see the responses to reviewer one question 1-3.*

*For the methylation calling, we compared the methylation information we identified with samples MDA-MB-231, GM12878, MCF-7, and MCF-10A we achieved at least 98% methylation agreement, we stated that in table S12.*

Major items

1. Given the enthusiasm amongst the LRS community for these technologies to identify all classes of variation, accurately, phased, genotyped and with methylation data, I was surprised to see the rates of mutation detection (SNV and SV), rates of phasing, and rates of phase errors lower or higher than expected - certainly lower than the abstracts conclusions would suggest. This manuscript would be much improved if these values were benchmarked to previous best practice approaches, to demonstrate that the performance of PRINCESS across all of its categories is indeed class-leading.

*We would like to thank the reviewer for the constructive suggestion. We compared our identified variants results to two other methods: Longshot for SNVs and CuteSV for SV calling.*

*PRINCESS SNV approach outperformed Longshot across all three technologies ONT, HiFi, and CLR as explained in the Figures 1-3.*

*Figure 1: SNVs comparison of F1, Precision, and recall between PRINCESS (blue) and Longshot (red) for ONT data.*

*Figure 2: SNVs comparison of F1, Precision, and recall between PRINCESS (blue) and Longshot (red) for HiFi data.*

*Figure 3: SNVs comparison of F1, Precision, and recall between PRINCESS (blue) and Longshot (red) for CLR data.*

*For SVs, we used the suggested parameters for CuteSV developers. While Sniffles performed better only in HiFi data, CuteSV achieved a better overall performance by ~3% as shown in Figures 4-6. Thus, we stick to using Sniffles as part of the PRINCESS pipeline.*

*Figure 4: SVs comparison of F1, Precision, and recall between PRINCESS (blue) and CuteSV (red) for ONT data.*

*Figure 5: SVs comparison of F1, Precision, and recall between PRINCESS (blue) and CuteSV (red) for HiFi data.*

*Figure 6: SVs comparison of F1, Precision, and recall between PRINCESS (blue) and CuteSV (red) for CLR data.*

2. The methylation results are hinted at on line 199 and then concluded to 'reveal the entire biological picture' on line 204. As presented, these claims are entirely unjustified. I recommend you summarise these findings in the main text, highlight specific examples where this information is useful (eg hypermethylation of a tumour suppressor gene previously reported to be important in these cell lines), and demonstration that these data are indeed phased, and that phasing has improved the interpretation of these data.

*We thank the reviewer for this comment. Indeed, we think that summarizing SNV, SV and methylation in a haplotype way does include a highly comprehensive if not a complete picture of genomic alterations and variation of the sample at hand. We have integrated a few citations to highlight the importance of phasing and SNV, SV and methylation. Nevertheless, proofing that these variant types matter is beyond the scope of this method paper.*

3. The PRINCESS GitHub homepage barely mentions methylation, so can the software actually process and phase methylation data?

*We have extended the documentation on the GitHub page. PRINCESS runs Nanopolish for methylation calling automatically. This is enabled by the flag “--methylation” and providing the directory for the fast5 files. We are currently also working on including Megalodon into the workflow.*

4. Phasing of SV and SNV: The conclusions on line 163-4 are unjustified. Phasing rates of 38-90% do not satisfy this reviewer as being high accuracy. These claims may be strengthened by comparing to state of the art methods, and appropriately reserved language. Adding specific examples where phasing helps resolve the pathogenicity/otherwise of alleles found to be in cis, or in trans.

*Phasing Structural Variations (complex or simple) is what is novel in our approach. The success rate of this step is not as high as SNV phasing due to the complexity of the task. It is often discussed that simple SV such as duplications hinder an accurate phasing of SNV in this region.*

*Across the capture data we phased 90% of the SNV and all but one SV. This is a simpler scenario compared to a genome wide approach. Here, for HS1011 the phasing rate for many SV is indeed reduced to 30%. This is without any conflicting read in the phasing process. This process can be improved with new methodologies, however, this is the first algorithmic implementation that phases all types of 50bp+ variants together with SNV.*

5. Accuracy of variant calling within the 193 difficult genes. Tackling these difficult to sequence genes is an important contribution of the paper. However, the quality and accuracy of the genotypes within these genes are not reported. The proportion of heterozygous sites within these difficult to sequence regions appears far higher than expected (ie very few homozygous alt sites) than typical genes. Line 226 suggests these 'does not seem to be affected' which is not supported by the data. In short - please provide evidence that these variants are real, preferably with orthogonal data (eg 2 or more LRS methods), and at a minimum, focussing on the quality attributes (AD, base quality, ...).

*Here we showcase that we are able to robustly map and identify variations across most of these genes. As you pointed out this does not work across all genes and there is sadly no truth set available for this sample. To circumvent this we assessed HG002 for these genes. The GIAB SNV benchmark set covers 96 (~50%) of the genes. Here PRINCESS achieves 90.55% F1 measure for SNV and indels (95.64% for substitutions only) and thus showing a great performance. As a reminder many of these genes are hard to assess as they are falling in segmental duplications or are collapsed segmental duplications. This could of course lead to the enrichment of heterozygous variations.*

*In addition, we also used Longshot for comparison across these 193 genes across HS1011. Figure 7 shows the number of identified variants for each tool as well as the distribution of variants, we can see that the ratio between heterozygous and homozygous variants are similar. In Figure 8 we merged the variants between the two methods and identified what is common and unique for each one. We see that ~74% of variants identified by PRINCESS are shared with Longshot and only ~26% of variants are unique to PRINCESS. Addationalt, Longshot did not identify Indels.*

*Figure 7: SNVs in the 193 medical genes identified using PRINCESS (blue) and Longshot (red), showing substitutions, indels, heterozygote, homozygote, and the overall count of variants.*

*Figure 8: SNVs in the 193 medical genes common (blue), Longshot unique (red), PRINCESS unique (yellow), and total variant (green) identified.*

6. HS1011 analysis: I found this difficult to follow, and I recommend that you focus on solving the diagnostic case in a succinct section, with a discussion the cis/trans alleles and how this information has helped diagnose the case. Are there any links to aberrantly methylated genes in CMT that your LRS data could shed light on in this case? The transition from candidate gene, SH3TC2, to the other 3 genes in Figure 5 could be improved; suggest you merge fig 4A and fig 5A together to speak to the diagnosis, and then Fig 4B,4C,5B-D in the same figure to speak to the exploration of difficult regions.

*We thank the reviewer for these suggestions and changed the figures and the order of the subsections accordingly.*

*We currently don’t know if there is an impact over methylation for this disease and cannot conclude something based on one single sample. Thats why we also didn’t stress methylation information for this sample.*

7. Translocations: 3.49% of SVs in HS1011 were translocations; I would expect this to be close to 0 in a germline case, unless that translocation affected the candidate gene, suggesting these are false positive calls, or perhaps low-level mosaic calls. Please investigate these.

*Other studies did not attempt to report translocations across full genomes. We intersected the identified translocations with transposons and other repeat coordinates from UCSC repeatmasker track. Overall, 79.72% of all the translocations overlap with these repeats. As you can see the majority with LINE and SINE elements where we speculate they are either copied or similar elements are inserted thus highlighted by the mapping of the long reads. 161 translocations are based in LTR regions. We included that now also in the main text and supplement table S19.*

*Table 1: Intersecting transposon count*

| *TRA intersect Transposon* | *Transposon Count* |
| --- | --- |
| *LINE* | *277* |
| *SINE* | *233* |
| *Satellite* | *195* |
| *LTR* | *161* |
| *Simple_repeat* | *45* |
| *Low_complexity* | *5* |
| *Retroposon* | *3* |

8. I found the discussion of rates of SV types relative to what's already known from short-read and LRS technologies to be difficult to follow. Eg on L241 and L244-245 speak to two separate contradictions in these data relative to 2 previous studies.

*The balance of insertion and deletions that we report follows the expectations based on several long read studies (e.g. GIAB). However, previously we showed that deletions were the vast majority due to errors for Oxford Nanopore, which is no longer the case. In addition, we highlighted the translocations (as also pointed out by you in the previous point) as either false positives (and thus might be ignored) or due to transposon events compared to the reference sequence. Neither translocations nor other complex events are often reported over long read studies.*

9. If showcasing the capabilities of PRINCESS, Figure 3 is underwhelming, as any short and long-read technology should easily resolve a 6Kb deletion, especially with two variants in trans overlapping the variant. What gene is affected here, and, based on the position relative to this transcript's TSS, you've missed an opportunity to investigate whether any regulatory elements are affected by this deletion, and whether they affect [publicly available] expression data for this transcript. I recommend you reconsider the elements in this figure to more holistically summarise the cancer data analysis.

*We have moved the figure to the supplement as this is just another showcase that PRINCESS also works correctly on capturing data. Nevertheless, it's worthwhile highlighting that PRINCESS can automatically identify these variants and phase them together. We have investigated this region and gene (XR_948351.2) if the deletion impacts the methylation of this region. We concluded it is not based on the figure below. Here the red line represents the sample (Mcf10a) with the deletion while the blue line MdaMb231, which did not carry this deletion.*

10. L432 - under what ethics was this patient consented? You indicate that this patient's data was shared to SRA. dbGaP or EGA seem a more secure choice for human genome data, so is SRA consistent with your ethics? What is the accession number?

*We deposited the data under bioproject PRJNA203659.*

11. The coverage subsampling analysis was hard to follow, with most of the justification for doing this appearing much later in the discussion. I recommend rewriting this section in a more proactive and justified way.

*We improved the text around the coverage assessment. However, we feel its an important point to show that PRINCESS works also at a lower coverage level. E.g. other manuscripts show their performance for e.g. SNV calling at ~60x data sets that are much more costly to obtain.*

Minor items

* This manuscript has dozens of grammatical and formatting errors throughout.

*We thank the reviewer and fixed the language in many places*

* Inconsistent spacing and commas before and within parentheses. Eg L116 twice, and page 6 in several spots

*We fixed that.*

* Supplementary vs Supplement throughout, followed by consistent capitalisation of Table or Figure or Section and spacing, eg L116, L124, L120, dozens more)

*Changes were made accordingly*

* Inconsistent spacing before citations, eg L63-65

*We changed spacing to be consistent. This unfortunately happens when downloading the google doc and for sure will be fixed in the proof version.*

* Figure 1C legend doesn't explain the role of coverage nor that subsampling analyses were performed

*Figure 1C shows the impact of increasing the coverage for the hamming errors (ie. phasing errors). As you can see with different coverage levels the phasing length (Figure 1B) as well as the error rate increases. Thus giving future scientists an estimation of what to expect from PRINCESS with different coverage levels.*

* Throughout, SNV and SV are mentioned, but not indels. If you have bundled indel performance into SNV performance than I recommend you make this clear at the start. Further, indel errors appear common in ONT data (and perhaps other LRS), so combining SNV with indels may explain why the reported SNV accuracy relative to NIST is lower than i would expect for only SNVs

*We thank the reviewer, we mentioned that we phase both substitutions and indels. “filtering and phasing of SNV (substitutions and indels) and SV together and” “so combining SNV with indels may explain why the reported SNV accuracy relative to NIST is lower than i would expect for only SNVs”*

*We reported the SNV and indels separately only in Supplementary Table S1.*

* L144 replace 'succeeded by'

*We changed it with “followed by”*

* Inconsistent use of commas and confusing use of two values, eg L147 - L151 or L159 are better examples of reporting multiple numbers

*Changed accordingly*

* L139 and L140, summarise what you found

*We summarized the results for phasing and different insert size HiFi data.*

* L143 and L154 both start with next; suggest a different approach.

*Changed accordingly*

* L163 'high rate of accuracy'; L206 'high concordance' - as mentioned above, this needs justification

* L174 is out of place; move to methods.

*Changed accordingly*

* L183 Flongual?

*We fixed the typo to Flongle*

* L195 - how large were these SVs?

*We have now adopted the line in the main text to make this clearer.*

*The SV are between 6 and ~156 Kb which was only mentioned in supplement table S16.*

|  | ***CHR*** | ***Original results*** | | | ***PRINCESS results*** | | |
| --- | --- | --- | --- | --- | --- | --- | --- |
|  |  | ***Start*** | ***Size*** | ***Gt*** | ***Start*** | ***Size*** | ***Gt*** |
| ***MDA-MB-231*** | *5* | *58,384,160* | *6,107* | *het* | *58,384,165* | *6,109* | *het* |
| ***MCF-7*** | *5* | *58,384,160* | *6,110* | *homo* | *58,384,165* | *6,109* | *homo* |
| ***MDA-MB-231*** | *7* | *154,600,417* | *7,569* | *het* | *154,600,191* | *7,852* | *het* |
| ***MCF-7*** | *7* | *154,600,417* | *7,569* | *homo* | *154,600,191* | *7,852* | *homo* |
| ***GM12878*** | *5* | *105,096,412* | *71560* | *het* | *105,096,412*** | *71,560* | *het* |
| ***GM12878*** | *6* | *78,257,496* | *69263* | *het* | *78,257,722* *** | *68,932* | *het* |
| ***GM12878*** | *8* | *39,374,556* | *155153* | *het* | *39,374,556*** | *155,153* | *het* |

* Tumorigenetic should be tumorigenic

*Changed accordingly*

* Performances should be performance

*Changed accordingly*

* L109 people should be researchers

*Changed accordingly*

* Variations should be variants

*Changed accordingly*

* Gene symbols should be italicised

*Changed accordingly*

* L269 … stars indicate…

*Changed accordingly*

* L283 was hard to follow

*Changed accordingly*

* L281-282 should be split and explained more clearly

* L287 how did you get 287 genes?

*We think the reviewer is referring to this sentence: “Using PRINCESS we identified 18,805 SNVs and 100 SVs across the 189 (one gene did not show any variant) of these genes that were covered, with an average of 101 SNVs per gene.”*

*We retrieved 192 out of the 193 genes from the ensembl annotation, two didn’t had sufficient converge and one gene did not show any SNV. Thus, we reported genes across 189. We tried to make this clearer now in the main text.*

* L299 clarify that these are hard to map from conventional short-read seq, or hard to map from LRS.

*HLA is in general very hard to resolve and this is generally accepted. One paper that points that out is the (cite GIAB HLA region). We have now added a sentence to explain this.*

* L308, L312 introduce the name of GBA gene with two different names?

*Changed accordingly*

* L328-L629 is unjustified

*We assume you mean the sentence “ Thus, enabling great insights into genomics ” we have removed this sentence. Clearly, PRINCESS enables a high level of comprehensiveness, but it is hard to prove this point across all data sets.*

* L334 - phasing and methylation is unsupported as noted above

*We respectfully disagree and show phasing of SV and methylation.*

* L350 typo

*Changed accordingly*

* L352 suggest to end with 'de novo variants'

*Changed accordingly*

* L3863-384 is repetitive

* L385 should be rephrased

*Changed accordingly*

**Second round of review**

**Reviewer 1**

The authors have improved the manuscript over the previous submission. The method will prove to be quite useful and the manuscript should be published. I do not recommend re-review, but there are statements that slightly overstate the novelty of the method, or what the method is doing.

"The deliverables from PRINCESS not only includes phased SNVs and indels, but further includes the first approach for SV phasing and inclusion of methylation phasing."

This is not true - assembly based methods produce phased SV callsets. I would recommend qualifying the statement to be regarding raw reads.

p 10: "Here, PRINCESS was able to phase 97.87% of the heterozygous SNVs and indels." Nothing is technically wrong with this statement, but because the primary phasing engine is WhatsHap, there will likely be others who view this statement as disingenuous. Because there is some special filtering of SNVs by PRINCESS, the case would be strengthened by modifying Figure 2A to show the gain over stock WhatsHap.

**Reviewer 2**

This is a much improved manuscript, particularly the discussion around figures 4 and 5. This remains a very dense publication, so in the hopefully final round of revision, I would advise on refining this in line with some of the suggestions below. It's very disappointing that I'm spending hours of my evening time pointing out basic things, like grammatical errors, the supp tables and figures are not in the right order, cross-references from text to supp figures are occasionally wrong.

Major corrections

*	The supp tables and figures are referred to out of order making it difficult to follow the results.

*	Abstract: 96.21 or 96.12? I don't see 97.92 mentioned anywhere in the text. Nor 85.6, or 90.91%. If these are important headline findings and are not discussed in the results section, then the abstract does not match the claims. If these numbers are buried in the supp tables, then you need to bring them to the main text.

*	P8 I gather that N50, switch error and hamming error are all interrelated features of phasing performance. I strongly recommend you introduce them as such, and consider how you shift from one to the other. Eg L19 links to Fig 2A but does not summarise the findings from this figure. The N50 data is in the supp, but not cited as such on L24, then switch error is quickly introduced. I recommend moving Supp Fig S5 and S6 into Fig 2, so it holistically summarises these parameters (much like the high quality Supp Fig 18) & consider revising how this section is introduced, and results are linked.

*	There are an overwhelming number of supp tables and figures. Some of the figures can be consolidated into one, as sub-panels, which I think would make the paper easier to digest. {Supp Fig 1,2,3} {5 and 6}, {7,8,9,10}, {13,14}, {15,16}.

*	P16 and throughout: the discussion of 189 vs 193 medical genes is clumsy and in some cases (eg the abstract), misleading: 2/193 genes had no coverage, so PRINCESS did not resolve anything in these genes. You should refer to them in the results, abstract, table S14 and discussion as 189 medically relevant genes, and in the methods, mention why the 193 genes from the original ref is reduced to 189 in your paper.

*	Re-use of public data: it wasn't clear at first review that most if not all of the data in this paper have been re-used. I have no issue with re-use of data, but it needs to be made clear throughout. I've added a few comments about the HS1001 data below, but I see that P7 L30 should be more explicit that these LRS data were generated by these publications. "… GIAB HG002 for SNVs, indels and SVs, /and used published LRS data from/ different coverage levels and seq tech…"

*	There is no statement about whether ethics was obtained to perform this research. This must be added.

*	There are some sentences linked to the wrong supp figure or table, so all of these must be checked before resubmission.

Minor recommendations

*	P4 L58: "Optionally,"

*	P7,L35 'supplement' should be 'supplementary' and throughout the paper.

*	P8, L12: which WhatsHap truth set? From NIST? Is this still on HG002 WGS data from previous sectoin?

*	P9, L14-19: I find this (and see comment on P20 L24-29) confusing. What here is disturbed? Suggest rephrasing this.

*	P10 referring to GM12878 and NA12878 is confusing. suggest you stick to NA1278

*	P10, L38 and throughout: hamming should be Hamming.

*	P10 L46, Why only 103 SNVs when you detected 87.44% of 226 variants in L43?

*	P10, L51, why are two percentages given?

*	P12,L16-21: you need to indicate that this was previously sequenced and cite the English et al study. You should link to the SRA dataset in the methods, and Table S15, and not on

*	Supp Figure 16 legend needs rewriting

*	P16 L29: Supp Fig S10 and Supp Table S15 don't appear to support this statement.

*	Fig 5B y-axis - percentage or proportion? I don't understand the y-axis, and P18, L21 suggests this has something to do with phasing? This needs fixing in text, figure legend and y-axis.

*	P19 L12 / Figure 5D - it's very difficult to see these three SVs from the screenshot. Please highlight them in the figure

*	P20L24-29: this new sentence doesn't make sense, please revise.

*	P22 L4 - 'on 193'

*	P22 L24 'Parkinson's Disease'

*	P25 L51 should be Table S15

*	P25 L53, re HS1001, as this is not data that you generated, it should not be listed here. Refer to it in Table S15 and in methods as noted above.

*	Several typos in the supp tables. S1,S6,S7,S9,S17,S18: SNP should be SNV. S2: 'percentage', 'correct GT', 'GT correctness'; S4: 'switch rate'; s5: 'precision'; s15 'insert size'; s16: 'hours'

*	All Supp Tables need a legend, some are missing. Many of the Supp Figure legends are overly brief.

*	Table S15 appears incomplete wrt file location, and is missing some datasets, including the HS1001 data, the Gilpatrick methylation data. As summarised here, there is insufficient information to know which relates to which data in the paper. Add more columns to describe the datasets more precisely. I recommend moving the info from supplement section 2.5 and 3.1 into Table S15.

**Authors Response**

**Point-by-point responses to the reviewers’ comments:**

Reviewer 1: The authors have improved the manuscript over the previous submission. The method will prove to be quite useful and the manuscript should be published. I do not recommend re-review, but there are statements that slightly overstate the novelty of the method, or what the method is doing.

1.	"The deliverables from PRINCESS not only includes phased SNVs and indels, but further includes the first approach for SV phasing and inclusion of methylation phasing."

This is not true - assembly based methods produce phased SV callsets. I would recommend qualifying the statement to be regarding raw reads.

*We thank the reviewer, we changed it accordingly:*

*"The deliverables from PRINCESS not only include phased SNVs and indels but further include the first alignment approach for SV phasing and inclusion of methylation phasing."*

2.	p 10: "Here, PRINCESS was able to phase 97.87% of the heterozygous SNVs and indels." Nothing is technically wrong with this statement, but because the primary phasing engine is WhatsHap, there will likely be others who view this statement as disingenuous. Because there is some special filtering of SNVs by PRINCESS, the case would be strengthened by modifying Figure 2A to show the gain over stock WhatsHap.

*We kinda disagree with this comment, however, we have performed this assessment across all three sequencing technologies on the highest coverage available. This was done to give the best possible case. We observed that omitting filtering led to an increase in both Hamming and Switch error as shown in Figure 1 and 2 below.*

Reviewer 2:

This is a much improved manuscript, particularly the discussion around figures 4 and 5. This remains a very dense publication, so in the hopefully final round of revision, I would advise on refining this in line with some of the suggestions below.

It's very disappointing that I'm spending hours of my evening time pointing out basic things, like grammatical errors, the supp tables and figures are not in the right order, cross-references from text to supp figures are occasionally wrong.

Major corrections

1.	The supp tables and figures are referred to out of order making it difficult to follow the results.

*Done*

2.	Abstract: 96.21 or 96.12? I don't see 97.92 mentioned anywhere in the text. Nor 85.6, or 90.91%. If these are important headline findings and are not discussed in the results section, then the abstract does not match the claims. If these numbers are buried in the supp tables, then you need to bring them to the main text.

*We thank the review, we changed the abstract according to the editorial guidelines*

3.	P8 I gather that N50, switch error and hamming error are all interrelated features of phasing performance. I strongly recommend you introduce them as such, and consider how you shift from one to the other. Eg L19 links to Fig 2A but does not summarise the findings from this figure. The N50 data is in the supp, but not cited as such on L24, then switch error is quickly introduced.

I recommend moving Supp Fig S5 and S6 into Fig 2, so it holistically summarises these parameters (much like the high quality Supp Fig 18) & consider revising how this section is introduced, and results are linked.

*We initially did not include N50 and switch error because the switching error is included in the hamming error plot and the N50 was mentioned. We now added info from the supplement table in the main text:*

*“HiFi data achieved the lowest switch (0.0040) and Hamming (0.0052) error rate (Supplementary Table S4 and Figure S5).”*

4.	There are an overwhelming number of supp tables and figures. Some of the figures can be consolidated into one, as sub-panels, which I think would make the paper easier to digest. {Supp Fig 1,2,3} {5 and 6}, {7,8,9,10}, {13,14}, {15,16}.

*We thank the reviewer for this suggestion, however, we don’t see much benefit in merging supplementary figures together. Instead of different numbers you still will have A,B,C,D..*

5.	P16 and throughout: the discussion of 189 vs 193 medical genes is clumsy and in some cases (eg the abstract), misleading: 2/193 genes had no coverage, so PRINCESS did not resolve anything in these genes. You should refer to them in the results, abstract, table S14 and discussion as 189 medically relevant genes, and in the methods, mention why the 193 genes from the original ref is reduced to 189 in your paper.

*We respectfully disagree. The 193 genes refer to the previous paper and thus it is almost like the name of the set. In the way we were not able to identify variants or coverage on the 3 genes is a form of assessment of Princess ability. Meaning, in 2 genes we failed to map reads on top.*

6.	Re-use of public data: it wasn't clear at first review that most if not all of the data in this paper have been re-used. I have no issue with re-use of data, but it needs to be made clear throughout. I've added a few comments about the HS1001 data below, but I see that P7 L30 should be more explicit that these LRS data were generated by these publications. "… GIAB HG002 for SNVs, indels and SVs, /and used published LRS data from/ different coverage levels and seq tech…"

*We apologize for this confusion. The data sets were taken from GIAB and we mention that throughout the result and “Availability of Data and Materials” section. For the capture data, it is also clearly highlighted at the beginning of the section.*

7.	There is no statement about whether ethics was obtained to perform this research. This must be added.

*We now added this section:*

*“Informed consent was obtained for individual HS1011 under protocol H-29697, which is approved by the Institutional Review Board at Baylor College of Medicine. This protocol provides consent to publish the detailed genomic information contained in this manuscript.”*

8.	There are some sentences linked to the wrong supp figure or table, so all of these must be checked before resubmission.

*We apologize again. We reordered and in the process checked this again.*

Minor recommendations

*	P4 L58: "Optionally,"

*Done*

*	P7,L35 'supplement' should be 'supplementary' and throughout the paper.

*Done*

*	P8, L12: which WhatsHap truth set? From NIST? Is this still on HG002 WGS data from previous sectoin?

*Yes, we initially described the comparison in the supplement, but moved it now to the method section.*

*	P9, L14-19: I find this (and see comment on P20 L24-29) confusing. What here is disturbed? Suggest rephrasing this.

*The alignment of reads can be impacted locally due to SV breakpoints. Thus, SNV calling can be impacted, and we observed in a couple of cases that the phasing of these SNV will be also disturbed. For example, near tandem duplications or deletions where the assumption of WhatsHap about the equal representation of both haploids is no longer the case.*

*	P10 referring to GM12878 and NA12878 is confusing. suggest you stick to NA1278

*The difference between GM and NA is the determination of the originating of the material. GM refers to cell lines directly whereas NA refers to the already extracted DNA.*

*	P10, L38 and throughout: hamming should be Hamming.

*Done*

*	P10 L46, Why only 103 SNVs when you detected 87.44% of 226 variants in L43?

*226 identified by GIAB, but the initial study reported only 103*

*	P10, L51, why are two percentages given?

*We changed it for clarity:*

*“we observed a slightly lower sensitivity (80.40% for all variants (SNVs and indels), 83.82% for SNVs only) “*

*	P12,L16-21: you need to indicate that this was previously sequenced and cite the English et al study. You should link to the SRA dataset in the methods, and Table S15, and not on

*We have sequenced this sample using Oxford Nanopore in this study. English et al. have sequenced that sample in the past with early Pacbio CLR data. We are already citing the two studies that first examined that sample and as we don’t use data from English et al. we didn’t cite him.*

*	Supp Figure 16 legend needs rewriting

*We changed this now to:*

*Fig. S16: Shows a 32kbp long deletion in sample MdaMb231 based on MinION sequencing data*

*	P16 L29: Supp Fig S10 and Supp Table S15 don't appear to support this statement.

*We agree with the reviewer, we corrected this.*

*	Fig 5B y-axis - percentage or proportion? I don't understand the y-axis, and P18, L21 suggests this has something to do with phasing? This needs fixing in text, figure legend and y-axis.

*On the Y axis we show the number of variants divided by the number of bases in the gene (ratio), the number of phase blocks are indicated by the color of SVs (triangle) and SNVs and indeed (circle).*

*We changed percentage to proportion.*

*	P19 L12 / Figure 5D - it's very difficult to see these three SVs from the screenshot. Please highlight them in the figure

*Done*

*	P20L24-29: this new sentence doesn't make sense, please revise.

*Changed accordingly:*

*“Phasing SV, however, remains challenging as SV also often leads to problems for SNV calling and thus SNV phasing due to alignment artifacts or simple assumption violations e.g. of het vs. hom ratios of SNV inside a duplication.”*

*	P22 L4 - 'on 193'

*Done*

*	P22 L24 'Parkinson's Disease'

*Done*

*	P25 L51 should be Table S15

*Done*

*	P25 L53, re HS1001, as this is not data that you generated, it should not be listed here. Refer to it in Table S15 and in methods as noted above.

*Sorry for the confusion, but it is data that we generated.*

*	Several typos in the supp tables. S1,S6,S7,S9,S17,S18: SNP should be SNV. S2: 'percentage', 'correct GT', 'GT correctness'; S4: 'switch rate'; s5: 'precision'; s15 'insert size'; s16: 'hours'

*We thank the reviewer, we changed these typos.*

*	All Supp Tables need a legend, some are missing. Many of the Supp Figure legends are overly brief.

*Done*

*	Table S15 appears incomplete wrt file location, and is missing some datasets, including the HS1001 data, the Gilpatrick methylation data. As summarised here, there is insufficient information to know which relates to which data in the paper. Add more columns to describe the datasets more precisely. I recommend moving the info from supplement section 2.5 and 3.1 into Table S15.

*Data have been added to the manuscript*
